# A scoping review of electroencephalographic (EEG) markers for tracking neurophysiological changes and predicting outcomes in substance use disorder treatment

**DOI:** 10.3389/fnhum.2022.995534

**Published:** 2022-10-17

**Authors:** Tarik S. Bel-Bahar, Anam A. Khan, Riaz B. Shaik, Muhammad A. Parvaz

**Affiliations:** ^1^Department of Psychiatry, Icahn School of Medicine at Mount Sinai, New York, NY, United States; ^2^Department of Neuroscience, Icahn School of Medicine at Mount Sinai, New York, NY, United States

**Keywords:** EEG, substance use disorder, event-related potentials (ERP), addiction, treatment, longitudinal, brain stimulation, recovery

## Abstract

Substance use disorders (SUDs) constitute a growing global health crisis, yet many limitations and challenges exist in SUD treatment research, including the lack of objective brain-based markers for tracking treatment outcomes. Electroencephalography (EEG) is a neurophysiological technique for measuring brain activity, and although much is known about EEG activity in acute and chronic substance use, knowledge regarding EEG in relation to abstinence and treatment outcomes is sparse. We performed a scoping review of longitudinal and pre-post treatment EEG studies that explored putative changes in brain function associated with abstinence and/or treatment in individuals with SUD. Following PRISMA guidelines, we identified studies published between January 2000 and March 2022 from online databases. Search keywords included EEG, addictive substances (e.g., alcohol, cocaine, methamphetamine), and treatment related terms (e.g., abstinence, relapse). Selected studies used EEG at least at one time point as a predictor of abstinence or other treatment-related outcomes; or examined pre- vs. post-SUD intervention (brain stimulation, pharmacological, behavioral) EEG effects. Studies were also rated on the risk of bias and quality using validated instruments. Forty-four studies met the inclusion criteria. More consistent findings included lower oddball P3 and higher resting beta at baseline predicting negative outcomes, and abstinence-mediated longitudinal decrease in cue-elicited P3 amplitude and resting beta power. Other findings included abstinence or treatment-related changes in late positive potential (LPP) and N2 amplitudes, as well as in delta and theta power. Existing studies were heterogeneous and limited in terms of specific substances of interest, brief times for follow-ups, and inconsistent or sparse results. Encouragingly, in this limited but maturing literature, many studies demonstrated partial associations of EEG markers with abstinence, treatment outcomes, or pre-post treatment-effects. Studies were generally of good quality in terms of risk of bias. More EEG studies are warranted to better understand abstinence- or treatment-mediated neural changes or to predict SUD treatment outcomes. Future research can benefit from prospective large-sample cohorts and the use of standardized methods such as task batteries. EEG markers elucidating the temporal dynamics of changes in brain function related to abstinence and/or treatment may enable evidence-based planning for more effective and targeted treatments, potentially pre-empting relapse or minimizing negative lifespan effects of SUD.

## Introduction

Substance use disorders (SUDs) constitute a growing yet preventable global epidemic with staggering costs for individuals and societies (Degenhardt et al., 2018; Rehm and Shield, 2019; Peterson et al., 2021) and involve high propensity for relapse (Sliedrecht et al., 2019), psychiatric comorbidities (Bahji et al., 2019; Castillo-Carniglia et al., 2019; Hunt et al., 2020), poor health outcomes (Degenhardt et al., 2018) and premature mortality (Martinez et al., 2020). As with other psychiatric disorders, SUDs are complex, arising over time from multiple cognitive, brain, genetic, and environmental factors whose interactions are not yet well understood (Gelernter and Polimanti, 2021; Heilig et al., 2021; Rawls et al., 2021; Ray and Grodin, 2021). Current models of SUD, mainly based on functional magnetic resonance imaging (fMRI; Devoto et al., 2020; Lin et al., 2020; Hill-Bowen et al., 2021; Klugah-Brown et al., 2021; Le et al., 2021; Sehl et al., 2021; Tolomeo and Yu, 2022) suggest chronic alterations in brain systems associated with reward processing (Luijten et al., 2017), salience attribution (Zilverstand and Goldstein, 2020), inhibitory control (Luijten et al., 2014; Le et al., 2021) and executive function (Quaglieri et al., 2020), which underpin disease-specific behaviors such as craving, compulsive drug-taking, and relapse (Zilverstand and Goldstein, 2020; Ceceli et al., 2022). However, to date there are no established brain-based biomarkers for precision SUD treatment monitoring and outcome evaluation, partly because these brain systems also show dysregulation in a range of other psychiatric disorders, making SUD-specific biomarker development and validation difficult (García-Gutiérrez et al., 2020; Niculescu and Le-Niculescu, 2022).

Approaches that are used for SUD treatment include pharmacological therapeutics (Klemperer et al., 2018; Volkow, 2020) and a range of non-pharmacological interventions: psychosocial (Dutra et al., 2008), contingency management (Prendergast et al., 2006; De Crescenzo et al., 2018; Bolívar et al., 2021), cognitive-behavioral therapy (Magill et al., 2019), cognitive bias modification (Boffo et al., 2019), group therapy (Coco et al., 2019), significant-other involvement (Ariss and Fairbairn, 2020; Schmit et al., 2022), and mindfulness (Cavicchioli et al., 2018; Korecki et al., 2020). More recently, non-traditional interventions such as neuromodulation (i.e., brain stimulation, Hauer et al., 2019; Zhang et al., 2019; Habelt et al., 2020; Bollen et al., 2022) and psychedelics (Tofoli and de Araujo, 2016; Romeo et al., 2021) have also received considerable attention as potential SUD treatments. Although the evidence of effective treatments for some SUDs is emerging, there are relatively few established or FDA-approved SUD treatments, which are mainly pharmacological (Volkow, 2020), along with problematic drug development pipelines (Negus and Banks, 2021), lack of new treatments for alcohol (Heilig et al., 2019) and cocaine (Brandt et al., 2021) use disorders, and no treatments for some SUDs (e.g., methamphetamine use disorder). Indeed, there are several challenges to effective and widespread SUD treatment, including high rates of dropout (Dutra et al., 2008) and relapse (Sliedrecht et al., 2019), as well as the lack of objective assessments of treatment efficacy and outcomes (Yücel et al., 2019). There are also large systemic gaps in the deployment of SUD treatment (Creedon and Cook, 2016; Rhee and Rosenheck, 2019; Mekonen et al., 2021; Saini et al., 2022) and the implementation of evidence-based treatments (Garner, 2009; Connery et al., 2020; Louie et al., 2021) that limit current knowledge in SUD research.

### EEG metrics provide access to brain dynamics

Electroencephalography (EEG) is a neurophysiological technique for measuring brain activity, and although much is known about EEG activity in acute (Fairbairn et al., 2021) and chronic (Hamidovic and Wang, 2019; Liu et al., 2022) substance use, knowledge regarding EEG activity in relation to longitudinal abstinence and treatment outcomes is sparse (Houston and Schlienz, 2018; Stewart et al., 2019b; Jurado-Barba et al., 2020). However, building on over half a century of concentrated EEG research, there is a growing interest in applying EEG approaches for SUD treatment research. Metrics derived from EEG have good test-retest reliability (Tenke et al., 2017; Ip et al., 2018; Malcolm et al., 2019; Roach et al., 2019; Vázquez-Marrufo et al., 2020; Cofresí et al., 2022), are highly heritable (Van Beijsterveldt and Van Baal, 2002; de Geus, 2010; Gilmore et al., 2010) and widely used in SUD (Parvaz et al., 2011; Almeida-Antunes et al., 2021; Zhang et al., 2021b; Liu et al., 2022), depression (de Aguiar Neto and Rosa, 2019), schizophrenia (Perrottelli et al., 2021), psychosis (Wang et al., 2022), neurodegeneration (Horvath et al., 2018), and pain (Ploner and May, 2018) research. High-quality EEG provides direct and millisecond-level access to regional and global neural activity (Sadaghiani et al., 2022), and indexes oscillatory communication (i.e., connectivity) within and between networks (Sadaghiani et al., 2022). In addition to self-report, behavioral performance, and clinical variables, EEG metrics can aid in detecting subtle risks or deficits (Houston and Schlienz, 2018; Jurado-Barba et al., 2020; Zhang et al., 2021b). Moreover, EEG methods are cost-effective and deployable outside of laboratories. With ongoing advances in technology and hardware development, EEG is an excellent choice for more effective and widespread integration of electrophysiological brain imaging in clinical, at-home and epidemiological (e.g., prospective cohort) assessments of SUD and treatment-related changes and outcomes.

Data extracted from EEG are usually analyzed in terms of event-related potential (ERP), spectral or time-frequency (oscillations/spectra; i.e., delta, theta, alpha, beta, gamma bands), and connectivity (e.g., coherence, phase-locking, graph-theory/networks) methods, either at the sensor (or groups of sensors) level or in terms of estimated neural sources. Here, we briefly overview ERP/EEG metrics to serve as a context for this review.

#### ERPs

A range of well-established ERP components exist that index specific neurocognitive processes, as reviewed in Woodman (2010), Luck and Kappenman (2011), Hajcak et al. (2019), Helfrich and Knight (2019), and Donoghue and Voytek (2022). ERPs are usually elicited by visual, auditory, or other sensory stimuli, with N and P in the ERP component’s name denoting negative and positive deflections, respectively, and the number reflecting their typical onset time in milliseconds relative to the onset of a stimulus, for example, N100 (or N1), P100 (or P1), N170, N200 (or N2), P300 (or P3), and N400. Other ERP components are named based on the process they are known to index, for example, mismatch negativity (MMN), error-related negativity (ERN), error positivity (Pe), or based on their temporal onset, e.g., late positive potential (LPP). In general, earlier ERPs, typically peaking before 300 ms, index earlier perceptual processing, whereas later ERPs are considered to reflect later cognitive and action-oriented processing (Luck and Kappenman, 2011). However, there is considerable heterogeneity and overlap in nomenclature for some (later) components, with some studies referring to a positive deflection in the P300 time windows as LPP Hajcak and Foti (2020), and others dividing long-duration components into subcomponents (Hajcak and Foti, 2020).

#### Oscillations/spectra and connectivity

Spectra [oscillations at delta (1–4 Hz), theta (4–7 Hz), alpha (8–12 Hz), beta (14–30 Hz), gamma (>30 Hz) frequencies] are usually analyzed in terms of resting (spontaneous) EEG or event-related changes in response to stimuli, as reviewed in Başar and Güntekin (2013), Herrmann et al. (2016), Karakaş and Barry (2017), Keil and Senkowski (2018), Karakaş (2020), Başar (2022), Cao et al. (2022), and Pavlov and Kotchoubey (2022). Brain oscillations are critical multiplex mechanisms considered to enact communication through coherence across distributed brain regions (Buzsáki et al., 2013; Fries, 2015; Schneider et al., 2021). Most frequency bands have clear spatial dominance and neural generators. These include occipital-parietal alpha (Quinn et al., 2021), medio-frontal and hippocampal theta (Cavanagh and Frank, 2014; Soltani Zangbar et al., 2020), and sensorimotor and basal ganglia beta (Khanna and Carmena, 2015; Asadi et al., 2022). Lower frequency bands (e.g., theta, alpha) are generally considered to serve as integrative large-scale rhythms (Fries, 2015), whereas work with event-related paradigms links vision with alpha (Sadaghiani and Kleinschmidt, 2016; Clayton et al., 2018), working memory with theta and alpha (Riddle et al., 2020; Pavlov and Kotchoubey, 2022) and reward with theta (Paul et al., 2020). Higher frequency bands such as beta (Khanna and Carmena, 2015) and gamma (Buzsáki and Wang, 2012; Başar, 2013) are usually linked to more local (regionally specific) and temporally brief activity, as well as, respectively, sensorimotor cognition (Engel and Fries, 2010; Spitzer and Haegens, 2017) and percept binding (Shin and Moore, 2019). See also van Ede et al. (2018), Schmidt et al. (2019), Ghiani et al. (2021), and Buergers and Noppeney (2022) for relevant reviews.

Connectivity metrics are considered critical for a better understanding of communication between brain regions, and are used widely across brain imaging modalities (EEG/MEG/fMRI). With EEG, resting and event-related (i.e., time-frequency) oscillations, as well as ERPs (Li et al., 2020), are used to study connectivity [e.g., *via* coherence, phase-locking, and graph-theory (Cao et al., 2022) between sensors, regions, or networks (Mahjoory et al., 2017; Sion et al., 2020; Sadaghiani et al., 2022)] to assess functional segregation and integration of brain activity.

### Previous SUD-EEG reviews

The following abbreviations are used throughout this review to specify various **SUDs** considered in the reviewed studies: (**AUD**, Alcohol Use Disorder; **CUD**, Cocaine Use Disorder; **CAN**, Cannabis Use Disorder; **MUD**, Methamphetamine Use Disorder; **NIC**, Tobacco/Nicotine Use Disorder; **OUD**, Opioid Use Disorder; **MIX**, Mixed, subsamples covering several different SUDs). See “Abbreviations” section for details.

Reviews of SUD-EEG research published since the year 2000 have summarized findings across various SUDs (Bauer, 2001a; Kouri and Lukas, 2001; Ceballos et al., 2009; Campanella et al., 2014; Houston and Schlienz, 2018; Stewart et al., 2019b; Zhang et al., 2021b; Liu et al., 2022), for specific SUDs, e.g., AUD (Hamidovic and Wang, 2019; Jurado-Barba et al., 2020; Agarwal et al., 2021), OUD (Wang et al., 2015; Motlagh et al., 2016; Ieong and Yuan, 2017; Stewart et al., 2019a), for specific ERPs (Campanella et al., 2014; Hamidovic and Wang, 2019; Fairbairn et al., 2021; Zhang et al., 2021b), for resting EEG (Liu et al., 2022), with an emphasis on longitudinal abstinence or treatment-related effects (Houston and Schlienz, 2018; Stewart et al., 2019b), and more recently, in regards to brain stimulation (Zhang et al., 2019; Habelt et al., 2020). Although most past EEG studies have been cross-sectional and focused on differences between SUD vs. non-substance-using controls, there is an emerging and much-needed set of SUD-EEG work emphasizing longitudinal treatment- and/or abstinence-related changes (Houston and Schlienz, 2018; Stewart et al., 2019b), paralleled by ongoing developments in SUD-MRI research (Moeller and Paulus, 2018; Hammond et al., 2019; Stewart et al., 2019b; Parvaz et al., 2022). Here, we provide a brief overview of past reviews to set a general context regarding current knowledge.

Reviews of work prior to 2001 pointed out that existing studies had focused mainly on AUD and indicated decreased oddball P3 in individuals with AUD compared to non-alcohol using controls (Bauer, 2001a; Kouri and Lukas, 2001). However, those studies were scarce, heterogeneous, and mainly cross-sectional (i.e., examining group differences between SUD samples and non-substance using controls), and lacked longitudinal within-subject assessments. Although many procedural and methodological issues remain in the field of SUD-EEG, meta-analyses confirm reduced and delayed oddball P3 in AUD (Hamidovic and Wang, 2019; Fairbairn et al., 2021) and in other SUD relative to non-using control groups, highlighting oddball P3 as a possible endophenotype or diagnostic biomarker for SUD. A 2021 meta-analysis reported that, compared to non-substance using controls, SUD samples showed higher P3 elicited by drug-related cues (i.e., drug paraphernalia, pictures of people using the drug) and lower N2 elicited during inhibitory control, i.e., No-Go trials of a Go/No-Go task (Zhang et al., 2021b). The review also noted that findings depended on SUD type, gender, and age as well as and preliminary evidence for recovery-related decreases in drug-cue P3 with abstinence or treatment (Zhang et al., 2021b). A 2014 review indicated, in AUD, decreased and delayed MMN and No-Go/oddball P3s, as well as decreased evoked potential (P50), and for AUD and CAN samples, decreased and delayed N400 elicited by incongruent sentences (Campanella et al., 2014). The authors also noted evidence for EEG-derived indicators of normalization (No-Go and oddball P3 in AUD, CUD, and OUD), abstinence (P50 in CUD), and recent substance use (MMN in AUD; Campanella et al., 2014). A 2018 review focused on several longitudinal studies and reported links to treatment outcomes including *abstinence* (decreased P3 to alcohol images in AUD), *relapse* (increased No-Go N170 to alcohol images in AUD, delayed oddball N2, decreased Eriksen flanker ERN), and *treatment non-completion* (decreased P3 using oddball and cue-reactivity tasks, in AUD, CUD, and NIC; Houston and Schlienz, 2018). A 2020 AUD-EEG review noted incomplete evidence for reduced Go/No-Go and oddball P3, reward (or loss) event-related theta, face-sensitive P1/N1/N170, and cue-reactivity LPP. The authors highlighted the paucity of evidence, except with oddball P3, for associations between most EEG metrics and SUD vulnerability, severity, outcomes, and course (Jurado-Barba et al., 2020). Similarly, another review emphasized existing longitudinal EEG studies only provide good evidence for P3 recovery with abstinence or treatment (Stewart et al., 2019b).

Early reviews of resting EEG studies (Bauer, 2001a) emphasized the nascent evidence for higher power in resting upper beta (~20–28 Hz) as a potential SUD marker based on a few existing AUD and CUD studies, along with the possibility of beta as a premorbid and heritable trait (Meyers et al., 2017). A recent major overview (Liu et al., 2022) of resting EEG studies noted that SUD groups, relative to non-substance-using controls, generally showed greater power at higher frequency bands (i.e., beta and gamma), whereas, with abstinence, there was greater power at lower frequency bands, i.e., delta and theta (Liu et al., 2022). Decreases in beta (or normalization to control group levels) were consistently associated with abstinence-related recovery in AUD, however, the existing evidence for recovery patterns was unclear or mixed for other frequency bands (theta, alpha) and SUDs (Liu et al., 2022).

### The current review

In this review, we focused on research articles published in the English language with adult SUD samples that included at least one earlier EEG assessment time-point (i.e., T1) and at least one later assessment (i.e., T2), with or without EEG. We excluded studies that were cross-sectional (e.g., SUD vs. non-substance using controls at one time point), or examined acute, withdrawal, or detoxification effects. Almost all studies involved some type of SUD treatment (e.g., being peri or post SUD rehabilitation, pharmacological, behavioral, or brain stimulation treatment), however, the time between the T1 and T2 varied considerably.

This review has both unique and overlapping features relative to past reviews. In particular, it has overlapped with past reviews of ERP abstinence and treatment predictors (Houston and Schlienz, 2018; Stewart et al., 2019b), abstinence-related resting EEG (Liu et al., 2022), and brain stimulation (Stewart et al., 2019b; Habelt et al., 2020). However, unlike previous reviews (Houston and Schlienz, 2018; Hamidovic and Wang, 2019; Stewart et al., 2019b; Habelt et al., 2020; Jurado-Barba et al., 2020; Liu et al., 2022), we emphasize both ERP and EEG/spectra, include any SUD type, and include studies from the last few years. Importantly, we differ from most past reviews in not including any cross-sectional studies, focusing only on prospective/longitudinal and pre/post treatment studies, and including both EEG/ERP and brain stimulation studies. Lastly, unlike most previous reviews, we also include the risk of bias quality ratings for the selected studies.

## Methods

### Study identification and selection

**Figure 1:**
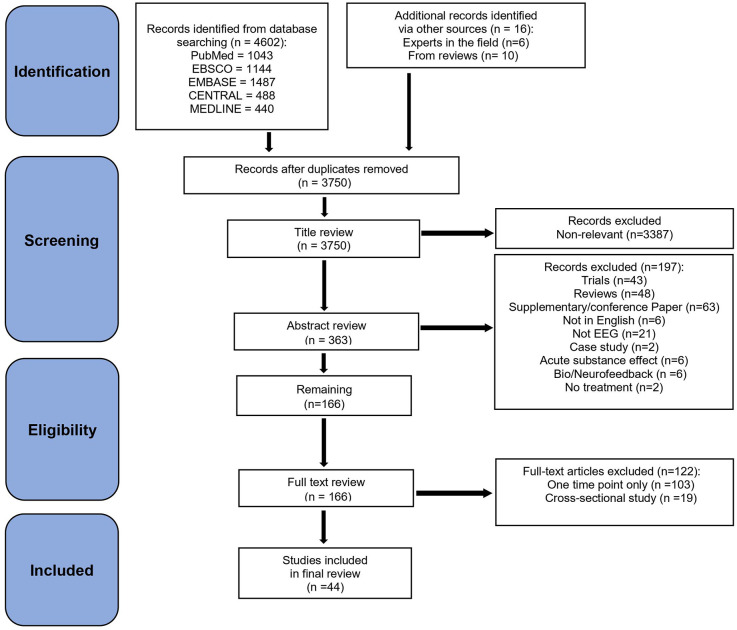
PRISMA flowchart.

The present scoping review followed the Preferred Reporting Items for Systematic Reviews and Meta-Analyses (PRISMA) guidelines (Moher et al., 2015) for study selection (Figure 1). Five databases (PubMed, Ebsco, Embase, Medline, and Cochrane Central) were searched by one reviewer (AAK) between March 14th and March 16th, 2022. Databases were searched for articles with terms that included [(EEG) AND (alcohol OR cocaine OR crack OR methamphetamine OR amphetamine OR opioids OR heroin OR hallucinogens OR MDMA OR ecstasy OR psilocybin OR ketamine OR sedative OR tobacco OR nicotine OR cannabis OR marijuana) AND (substance use disorder OR addiction OR dependence OR drug abuse OR abstinence OR cessation OR treatment OR recovery OR relapse)].

The inclusion criteria consisted of: (a) articles in English; (b) published on or after the year 2000; (c) conducted on adult (>18 years old) participants during an awake state; (d) included empirical data; (e) used ERP, spectral (resting-state) and associated techniques; (f) included a SUD sample; and (g) included at least one earlier EEG time-point and at least two time-points overall. We excluded: (a) studies that focused on the acute, withdrawal, or detoxification effect of substance use on EEG; (b) in which multiple influences could not be separated (e.g., main emphasis on depression, or use of a substance in the study, e.g., alcohol or cigarettes; (c) case studies; (d) reviews; (e) conference or supplementary reports, and (f) studies that focused on behavioral addictions (e.g., gambling/internet addiction), pain, genetic/environmental factors, and sleep. Although reflecting a promising possible future SUD treatment approach, bio/neurofeedback studies (*N* = 6) were excluded due to not using EEG as a Time 1 or Time 2 dependent variable (but only for the treatment), and standing issues in the neurofeedback research field regarding terms of evidence sparsity, methods, and clinical validity. See Dousset et al. (2020), Trambaiolli et al. (2021), Fernández-Álvarez et al. (2022), and Lima et al. (2022) for recent neurofeedback reviews.

The database search provided an initial set of 4,602 articles. We added 16 more articles that were obtained from experts in the field and from studies cited in previously published review articles. After duplicates were removed manually, 3,750 articles remained that were then screened for eligibility. Title, abstract, and full-text screening were performed by AAK and TSB independently. Consensus on any discrepancies was reached through subsequent group discussion, with all authors reaching a consensus on which articles should be included. The first screening was done by reviewing article titles, resulting in 3,430 articles removed because of irrelevance, and 320 articles retained. A second screening of full abstracts led to 154 articles removed, and 166 retained. A third screening was then conducted based on the full text of these 166 studies, and 122 articles were removed mainly because they either did not have two time-points (starting with at least one EEG at T1 and either EEG or any other variable at T2) or they were cross-sectional studies between SUD vs. non-substance using controls and/or SUD sub-classes. Finally, the remaining 44 articles were included in the present review.

### Data extraction

Two reviewers (AAK, TSB) performed the extraction of data from the studies. Table 1 summarizes the main details collected per study, including author, year, SUD sample type, sample size per group at T1 and T2, type and details (dose/session) of treatment, EEG methods (type of EEG metric (ERP or Rest, ERP metrics, task(s), number of channels). The main findings were extracted by TSB and RBS. Additional information per study is summarized in Supplementary Table 1, including age, sex, education, ethnicity, time and abstinence details, main criteria/interview for SUD determination, duration of use, and usage rates.

**Table 1:** Main summary of study characteristics.

**First author and year**	**SUD and study type**	**Sample size at T1 and T2**	**Treatment details**	**Dose/Sessions details**	**Time window**	**EEG** **Type** **Task** **Channels**	**Main findings**
**STUDIES OF ABSTINENCE and TREATMENT-AS-USUAL OUTCOMES**							
***Event-related potentials (ERPs)***							
Batschelet et al. (2021)	AUD LONG	T1: AUD: 59 Controls: 20 T2: AUD: 51 Relapse: 25 Abstinent: 26 Controls: 20	Hospital inpatient (8–12 weeks)	**	3 months	ERP: N2, P3 GNG, drug cues Channels: 64	**T1:** No main ERP effects distinguishing Relapse vs. Abstinent groups at T2 **T1:** Trend: ↑ P3 (alcohol vs. neutral) in Relapse group only **Other: T1:** ↑ craving linked to ↑ frontal N2 (No-Go Alcohol vs. Neutral) with anterior cingulate, medio-frontal, inferior parietal, and temporal gyrus sources
Campanella et al. (2020)	AUD LONG	T1: 40 T2: 40 T3: Relapsed: 25 Abstinent: 15	Hospital inpatient	Diazepam + craving medication + group therapy (4 weeks)	T3: 3 months	ERP: P3 visual oddball, GNG, drug cues Channels: 32	**T1 to T2:** Trends: ↑ P3d (No-Go, alcohol contexts) in Abstinent group only; Other: Trends: T1 to T2: ↓ P3d (cue-reactivity, non-alcohol context) for Abstinent group only; ↓ P3d (cue-reactivity, overall) for Relapse group only.
Matheus-Roth et al. (2016)	AUD LONG	T1: AUD: 30 Controls: 31 T2: Relapse: 12 No-Relapse: 11	Hospital inpatient (at end of treatment)	**	3 months	ERP: N170, P100 GNG, drug cues Channels: 32	**T1:** ↑ N170 (No-Go, alcohol cue) linked to Relapse vs. No-Relapse: at T2 **Other:** ↑ P100 latency at T1 linked to ↑ depressive symptoms at T2; ↑ Abstinence linked to ↓ depression
Petit et al. (2014)	AUD LONG	T1: AUD: 27, Controls: 27 T2: Relapse: 13 No-Relapse: 14	Hospital inpatient detox program	**	3 months	ERP: N2, P3 GNG Channels: 32	**T1:** ↑ P3d (No-Go vs. Go) linked to No-Relapse vs. Relapse at T2 (also faster relapse) **Other: T1:** P3d predicted Relapse above and beyond age, family history, craving; P3d and impulsivity together predicted Relapse; Clinical variables did not distinguish Abstinence vs. Relapse
Petit et al. (2015)	AUD LONG	T1: AUD: 39 Controls: 29 T2: Relapse: 19 No-Relapse: 20	Hospital inpatient Benzodiazepines	**	3 months	ERP:P3 oddball, drug cues Channels: 32	**T1:** No main group effect (Relapse vs. No-Relapse) **Other:** Trend: T1 ↓ P3 (alcohol vs. non-alcohol cues) in No-Relapse group only, with inferior medial and temporal gyrus sources,; EEG best predictor of Relapse relative to craving and depression
Marhe et al. (2013)	CUD LONG	T1: CUD: 49 Controls: 23 T2: Relapse: 31 No-Relapse: 18	Residential	**	3 months	ERP: ERN Eriksen flanker Channels: 32	**T1:** ↓ ERN linked to ↑ use in last 30 days at T2 **Other:** ERN strongest predictor of cocaine use relative to clinical variables, and ERN linked to severity and craving
Parvaz et al. (2017)	CUD LONG	T1: CUD: 19 Controls: 18 T2: same	Community sample	**	6 months	ERP: LPP affective/drug cues Channels: 64	**T1 to T2:** ↑ LPP (Pleasant vs. Drug) **Other:** T1 to T2 ↑ LPP (Pleasant vs. Drug) effect predicted ↓ craving (from T1 to T2)
Lespine et al. (2022)	NIC LONG	T1: 120 T2: Group 1: 44 Group 2: 41 Group 3: 35	Community sample Cessation interventions: App, contingency, health service	**	12 months	ERP: N2, P3 SST, MID Channels: 64	**T1:** Correct stop: ↑ left central/frontal N2 latency, ↑ fronto-lateral P3, ↓ left central and right frontal P3 Failed Stop: ↓ right central ↑ right posterior and fronto-lateral; Linked to longer Time to Relapse **T1 to T3:** Correct stop: ↓ frontopolar and left posterior late ERP; Failed Stop: ↑ right-central P3; Linked to longer Time to Relapse **Other:** The most predictive variables of time to relapse were psychological and emotion state variables.
Luijten et al. (2016)	NIC LONG	T1: 72 T2: Relapse: 37 No-Relapse: 25	Community sample Varenicline Bupropion, Patch, Counselling	**	12 weeks	ERP: P3, LPP, N2, ERN, Pe cue-reactivity, GNG, Eriksen flanker Channels: 34	**T1:** ↓ P3 ↑ N2 (GNG) linked to Relapse vs. No-Relapse at T2 **Other:** Trends: P3 to smoking behavior, Pe to Relapse, ERN to behavior; Relapse prediction (with ERPs included) effects stronger in female participants
Versace et al. (2012)	NIC LONG	T1/T2/T3/ T4/T5/T6: Cluster 1: 99/70/50/ 55/53/40 Cluster 2: 81/45/32/ 30/28/20	Community Behavioral counseling (Bupropion or Varenicline or placebo)	10 week Drug trial	24 weeks	ERP: LPP affective/drug cues Channels: 128	**T1:** Pattern of ↓ LPP (Pleasant cues) vs. ↑ LPP (Pleasant cues) linked to Relapse vs. Abstinence (at 10 and 24 week only) **Other:** Cluster 1: ↑ LPP (Pleasant cues); Cluster 2: ↓ LPP (Pleasant cues)
Haifeng et al. (2015)	MUD LONG	T1: MUD: 26 Controls: 29 T2: MUD: 24 Controls: 25 T3: MUD: 24 Controls: 23	Compulsory residential	Relapse prevention education, exercise, physical labor	T2: 3 months T3: 6 months	ERP: P3 Stroop using drug/neutral words Channels: 60	**T1 to T2:** ↓ left anterior P3 (drug words) **T1 to T3:** ↓ left anterior P3 (drug words) **Other:** ↓ P3 linked to ↓ craving at T2
Lubman et al. (2009)	OUD LONG	T1: OUD: 33 Controls: 19 T2: Relapse: 12 No-Relapse: 19	Methadone Buprenorphine	Methadone: No-Relapse: 55.2 (43.7) Relapse: 46.7 (20.8) mg/day Buprenorphine: No-Relapse: 7.3 (5.8) Relapse: 12.5 (12.3) mg/day	6 months (3.9–8)	ERP: P3 affective/drug cues Channels: 3	**T1:** ↓ startle-elicited P3 attenuation (pleasant vs. drug) linked to ↑ recent heroin use at T2* **Other:** EEG weak predictor of T2 use relative to self-reported valence ratings of pleasant images
Anderson et al. (2011)	MIX (AUD, CUD, OUD) LONG	T1: 35 T2: Completers: 22 Discontinuers: 13	Residential	3 months	≥80 days*	ERP: P3a auditory perseveration Channels: 64	**T1:** ↓ midline P3a linked to treatment discontinuation at T2 [check what time in narrative] **Other:** central P3a only predictor of treatment outcome relative to other variables (age, education, severity, IQ)
Fink et al. (2016)	MIX (CUD, MUD, OUD) LONG	T1: 123 T2: Completers: 98 Discontinuers: 25	Incarcerated sample Counseling, Relapse Prevention, Substance Expectation Therapy	≤12 sessions	≤12 weeks*	ERP: P3a, N2 GNG, visual distractor, visual oddball Channels: 64	**T1:** ↓ P3a (oddballs) and ↑ N2 (distractors) linked to treatment discontinuation at T2
Steele et al. (2014)	MIX (CUD, MUD, OUD) LONG	T1: 89 T2: Completers: 68 Discontinuers: 21	Incarcerated sample Counseling, Relapse Prevention, Substance Expectation Therapy	≤12 sessions	≤12 weeks*	ERP: P2, ERN/Ne, P2, Pe GNG Channels: 64	**T1:** ↓ P2, ↓ ERN/Ne, and ↑ Pe linked to treatment discontinuation at T2 **Other:** P2 and Pe strongest predictors of treatment discontinuation relative to all clinical variables; Principal component EEG metrics better in predicting treatment discontinuation relative to ERPs
Wan et al. (2010)	MIX (AUD, CUD, MUD, OUD, CAN) LONG	T1: 44 T2: Completers: 26 Discontinuers: 15	Residential	**	≤6 months	ERP: P3 visual oddball Channels: 64	**T1:** ↓ parietal P3 linked to treatment discontinuation at T2 ERP better predictor of treatment discontinuation than clinical variables.
***Spectral studies***							
Januszko et al. (2021)	AUD LONG	T1: 73 T2: Abstinent: 18 Not-Abstinent: 16	Residential	**	12 months median (9–14)	Rest: EC, 300 s Channels: 64	**T1:** ↑ δ left dorsolateral prefrontal connectivity with left inferior parietal lobe; right inferior frontal junction connectivity with right anterior insula; linked to Abstinence at T2 **Other:** δ inferior frontal junction connectivity with anterior insula correlated with personality and age of smoking onset
Saletu-Zyhlarz et al. (2004)	AUD LONG	T1: AUD: 22 Controls: 22 T2: Abstinent: 11 Relapse: 11 Controls: 22	Relapse prevention therapy + flupentixol	10 mg injection or placebo every 2 weeks	6 months	Rest: EC, 240 s Channels: 19	**T1:** *Absolute power*: ↑ δ right frontal, ↓ α2 frontal, central, temporal, right parietal, ↓ β1 central, parietal, left frontal-temporal, ↓ β2 left frontal-parietal. *Relative power*: global ↑ δ / θ; Linked to Abstinence at T2 **T2:** *Absolute power*: ↓ frontal δ / θ ; ↑ β3 right frontal, left temporal ; ↑ β4 right frontal, left occipital-temporal, ↑ β5 frontal, right occipital, left parietal; *Relative power*: ↓ α1 frontal-right occipital, ↑ β global; linked to Relapse at T2
Levin et al. (2007)	CUD LONG	T1: CUD: 20 Controls: 20* T2: CUD: 8 T3: CUD: 6	Clinical research ward	**	34.7 (24.4; 8–77) days	Rest: EC, 180 s Channels: 8, 16	**T1 to T3:** ↑ left temporal β1; ↑ right temporal δ; Trend ↑ β2 **Other:** Trends: ↓ left frontal delta to beta2, ↑ right central and occipital delta with ~2 weeks abstinence
Prichep et al. (2002)	CUD LONG	Cluster 1/Cluster 2/ Cluster 3: T1: 9/25/23 T2: >25 weeks: 2/20/8 T2:≤25 weeks: 7/5/15	Residential	**	25 weeks Median	Rest: EC, 1200 s Channels: 19	**T1:** 3 EEG Clusters; Cluster 1: ↑ relative θ, max posterior; ↑ relative β; Cluster 2: ↑ Absolute and relative α; Cluster 3: ↑ Absolute α and relative β; also occipital α and β coherence (max for Cluster 1), and frontal α coherence (Cluster 2)
Venneman et al. (2006)	CUD LONG	T1: 25 T2: Completers: 14 Non-Completers: 5	Hospital inpatient and outpatient	Selegiline 10 mg/day or placebo for 8 weeks Individual and Group therapy*	8 weeks	Rest: EC, 20–32 s Channels: 19 Cordance metric	**T1:** Treatment completion: Cordant 85%, Discordant: 15% completion, 25% of dropouts, 50% of removed patients **Other:** Cordant Week 2, 92%, final attrition 15%, Discordant Week 2, 83% and final attrition 100%. EEG better predictor of treatment completion than clinical and toxicology-screen variables
Allsop and Copeland (2016)	CAN LONG	T1: 10 T2: same	Community based non-treatment seeking	Contingency program	2 weeks	Rest: EC, 120 Channels: 14	**T1 to T2:** No effect **Other:** ↑ frontal β from T1 to T2 linked to ↑ age of first CAN use and age β independent predictor of Abstinence relative to other study variables
Herning et al. (2003)	CAN LONG	T1: CAN: 29 Controls: 21 T2: CAN: 29	Clinical research ward	**	25–27 days	Rest: EC, 180 Channels: 16	**T1 and T2:** ↓ θ and α1 **Other:** Trends:↓ β1 and β2
Herning et al. (2008)	CAN LONG	T1: CAN-short: 56 CAN-long: 19 Controls: 33 T2: CAN-short: 42 CAN-long: 17 Controls: 33	Clinical research ward	**	1 month	Rest: EC, 180 s Channels: 16	**T1 and T2:** ↓ posterior α2 and β2; ↓ occipital α peak, in CAN-long group only **Other:** EEG strongest predictor relative to age and clinical variables
Bauer (2001b)	MIX (AUD, CUD, OUD, benzodiazepines LONG	T1: SUD: 107 Controls: 22 T2: Relapse: 48 No-Relapse: 59 Controls: 22	Residential	**	6 months	Rest: EC, 300 s Channels: 15	**T1:** ↑ relative frontal β2 linked to Relapse vs. No-Relapse **Other:** EEG strongest predictor (relative to family history, severity); ↑ β2 in CUD/AUD relative to the OUD/polydrug participants
**TREATMENT STUDIES**							
***Brain stimulation***							
da Silva et al. (2013)	AUD Pre-Post STIM	T1: Sham: 7 Active: 6 T2: Relapse: Sham: 1 Active: 4 No-Relapse: Sham: 6 Active: 2	tDCS	2 mA, 20 min 1 session per week	T2: 5 weeks T3: s4 weeks	ERP: LPP alcohol/neutral cues Channels: 32	**Pre/Post:** ↓ LPP (alcohol and neutral cues) **Other:** ↓ Depression ↓ craving ↑frontal and orbitofrontal activity (Neutral cues) ↑ frontal, orbitofrontal and dorsolateral prefrontal cortex, and ↓ anterior cingulate activity (alcohol cues)
Del Felice et al. (2016)	AUD Pre-Post STIM	T1: Sham: 9 Active: 8 T2: same	Hf-rTMS	10 Hz 10 min* 2 sessions per week	2 weeks	Rest: EC, 300 s Channels: 32	**Pre/Post:** ↓ γ **T1:** β greater than δ **T2:** δ greater than γ **Other:** ↑ stroop and Go-No-Go scores ↓ depression/somatization/sleep symptoms no EEG link with craving or use positive correlation of central α and θ with depression/somatization
Nakamura-Palacios et al. (2012)	AUD Pre-Post STIM	T1: Lesch I: 16 Lesch II: 7 Lesch III: 4 Lesch IV: 12 T2: same	STIM tDCS	1 mA 10 min 2 sessions (1 active, 1 sham)	1 week	ERP: P3 auditory cue-reactivity Channels: 32	**Pre/Post:** Lesch IV group: ↑ frontal P3, alcohol cues; ↓ centroparietal P3, alcohol cues; opposite P3 patterns for Lesch II **Other:** ↑ frontal assessment scores for Lesch IV
Naim-Feil et al. (2022)	AUD Pre-Post STIM	AUD: 11 Controls: 16	rTMS	inhibitory paired-pulse TMS ~30 min 1 session	1 session	Stimulation-evoked EEG inhibition Channels: 24	**Pre/Post:** Left frontal STIM target: ↑ clustering coefficient and ↓ global/local efficiency **Other:** Links between ↓ mean degree and clustering in relation to ↑ severity of past use
Conti et al. (2014)	CUD (crack) Pre-Post STIM	T1: Sham: 6 Active: 7 T2: Sham: 3 Active: 6 T3: Sham: 1 Active: 5	tDCS	2 mA 20 min 5 sessions	T1: 1 session T3: 10 days	ERP: P3 GNG, drug/ neutral cues Channels: 32	**Pre/Post T1:** ↑ left P3 (neutral) ; ↓ left P3 (drug cues); ↑ right P3 (drug cues); dorsolateral prefrontal sources **Pre/Post: T1 vs. Session 5:** ↓ frontal, orbitofrontal, anterior cingulate activity (neutral cues); ↑ frontal, orbitofrontal, dorsolateral prefrontal, anterior cingulate activity (drug cues)
Conti and Nakamura-Palacios (2014)	CUD (crack) Pre-Post STIM	T1: Sham: 6 Active: 7 T2: same	tDCS	2 mA 20 min 1 session	1 session	ERP: P3 GNG, drug/ neutral cues Channels: 32	**Pre/Post:** ↓ N2 (drug cues); anterior cingulate sources
Pripfl et al. (2014)	NIC Pre-Post STIM	T1: 11 T2: same	Hf-rTMS	10 Hz 12 min 2 sessions (1 active, 1 sham)	1 week	Rest: EC, 180 s Channels: 9	**Pre/Post:** ↓ δ and α; ↑ γ **Other:** ↓ craving
Chen et al. (2021)	MUD Pre-Post STIM	T1: Sham: 22 Active: 35 T2: Sham: 19 Active: 30	rTMS	3-pulse 50-Hz bursts (~ 200 s) 5 min 20 sessions 5 sessions a week	4 weeks	ERP: N100 addiction stroop Channels: 64	**Pre/Post:** ↓ fronto-central β **Other:** Trends: ↑ N1 amplitude (methamphetamine vs. neutral cues); N1 change* positively correlated with T2 cue-induced craving
Khajehpour et al. (2022)	MUD Pre-Post STIM	T1: Sham: 20 Active: 22 T2: same	tDCS	2 mA 20 min 1 session	1 session	ERP: P3, LPP cue-reactivity Rest: EO, 120 s Channels: 62	**Pre/Post:** ↓ P3 (drug vs. neutral cues), only in post-STIM group vs. sham **Other:** STIM P3 effect linked to pre-STIM ↓ resting-state network efficiency at right superior frontal gyrus, supplementary motor area, Heschel's gyrus and left medial frontal, inferior frontal, inferior occipital, superior parietal, angular gyrus regions
Zhang et al. (2021a)	MUD Pre-Post STIM	T1: Sham: 20 Active: 20 T2: Sham: 16 Active: 18	iTBS	3 50 Hz pulse trains ~4 min* 20 sessions 2 sessions a day	15 days*	Rest: EC, 600 s Channels: 128	**Pre/Post:** ↓ central and left central-parietal δ **Other:** ↓ δ linked to treatment-related ↑ in 3-back working memory performance ↑ working memory performance
Mostafavi et al. (2022)	OUD Pre-Post STIM	T1: 30 T2: Group A: 10 Group B: 10 Sham: 10	tDCS	2 mA 20 min 10 sessions	10 days	Rest: EC, 180 s Channels: 19	**Pre/Post:** *Left anode*: ↓ bilateral frontal and central δ, bilateral frontal and right-lateralized occipital-parietal β2; *Connectivity*: ↑ right fronto-central and left fronto-temporal δ, fronto-central and right fronto-temporal β, parieto-temporal α **Pre/Post:** *Right anode*: ↓ left occipital alpha and β/β1; *Connectivity*: ↑ left frontal-parietal θ and α, ↓ right centro-frontal δ
Nakamura-Palacios et al. (2016)	MIX (AUD, CUD) Pre-Post STIM	T1: AUD: 22 Sham: 14 Active: 8 CUD: 9 Sham: 3 Active: 6 T2: same	tDCS	2 mA 20 min 5 sessions	5–10 days*	ERP: P3 Channels: 32	**Pre/Post:** ↑ P3 with ventromedial prefrontal sources linked to Abstinence vs. Relapse (during and after treatment)*
***Pharmacological and behavioral treatments***							
Brown et al. (2020)	AUD Pre-Post BEH+ STIM	MBRP+Active: 36 MBRP+Sham: 32	STIM tDCS MBRP	≥ 8 sessions	2–3 months	ERP: LPP drug cues Channels: 128	**Pre/Post:** ↓ LPP (alcohol cues but not neutral cues) linked to MBRP; ↑ LPP linked to STIM **Other:** More MBRP sessions led to ↓ craving
Martinez-Maldonado et al. (2020)	AUD Pre-Post BEH	T1: A-CBM: 10 N-CBM: 12 No-Intervention: 11 T2: same	A-CBM	8 sessions of A-CBM and N-CBM 4 sessions of No-Intervention	1 week	Rest: EC, 180 s Channels: 64	**Pre/Post:** A-CBM: ↑ α connectivity, and avoidance to alcohol-related cues; **T1 to T2:** Trend: ↑ anterior-central beta connectivity **Other:** ↑ posterior α, all conditions and groups; anterior/posterior β effects; β inversely correlated with baseline avoidance of alcohol-related cues
Cinciripini et al. (2017)	NIC Pre-Post Drug	T1: 180 T2: Varenicline: 58 Bupropion: 59 Placebo: 63	Varenicline Bupropion Smoking cessation counseling (12 weeks)	6 in-person sessions 4 phone sessions Varenicline: 0.5 mg/day for 1–3 days, 0.5 mg 2× a day for 4–7 days, and 1 mg 2× a day thereafter; Bupropion: 150 mg/day for 1–3 days, 150 mg 2× a day thereafter)	6 months	ERP: LPP Affective/drug cues Channels: 128	**T1:** Pattern of LPP Pleasant>cigarette (vs. cigarette>Pleasant) linked to Abstinence at T2 **Other:** Change off benefit at end of treatment and 3-month follow-up: 99%/99% with varenicline; 67%/43% with bupropion-
Li et al. (2017)	NIC Pre-Post BEH	T1: 42 T2: same	Hypnosis	1 session	1 h	Rest: EC, 480 s Channels: 64	**Pre/Post:** ↑ δ; ↓ α and β; **Other:** ↑ δ posterior bilateral coherence linked to ↓ craving; trend: ↑ θ;
Macatee et al. (2022)	CAN Pre-Post BEH	T1: DTI: 30 HVC: 30 T2: DTI: 30 HVC: 27	DTI or HVC	2 (1 h) sessions over 1 week	4 months	ERP: LPP, affective/drug cues Channels: 64	**Pre/Post:** No main effect on LPP **Other:** ↑ LPP (post-stressor, drug cue) linked to ↑ severe CUD and ↑ chronicity at 4 months ↑ LPP for drug and threat vs. neutral cues
Motlagh et al. (2018)	OUD Pre-Post Drug	T0: OUD: 35 Controls: 19 T1: OUD: 31 T2: OUD: 17	Methadone Maintenance Therapy	Methadone: Initial 30 mg/day titrated to ~70–85 mg/day	10 weeks	ERP: MMN, P3 Rest: EC, 300 s Channels: 19	**Pre/Post:** Delayed P3. No effect on amplitudes. Trends: ↓ frontal β, ↑ temporal δ and α, ↑ global θ **Other:** ↑ θ correlated with ↑ withdrawal (T3*); ↓ withdrawal with treatment
Robinson et al. (2022)	MIX (NIC, AUD) Pre-Post Drug	T1: 101 T2: Topiramate- low: 29 Topiramate- high: 36 Placebo: 36	(Topiramate: high/low dose, 5 weeks) Brief behavioral compliance enhancement treatment (18 weeks)	Topiramate: Low dose: ≤125 mg/day High dose: ≤ 250 mg/day (Titrated first 5 weeks, maintained 6th to 18th week)	8 months	ERP: LPP affective/drug cues Channels: 128	**T1 to T2:** ↓ LPP amplitudes (affective and drug cues, but not neutral cues) for high-dose vs. low-dose Topiramate **Other:** LPPs did not mediate medication effects on post-quit use, reinforcement, craving, or withdrawal

The main findings column summarizes selected study details, focusing mainly on EEG-related significant effects and/or trends. All ERP effects are in terms of amplitude and all spectral effects are in terms of absolute power unless otherwise noted. Up or down arrows refer to increases/greater/higher and decreases/less/lower in the specified metric. For negative-going ERPs (e.g., N2) up means more negative and down means less negative. Numeric details are usually provided as counts or as percentages. Means (averages) are followed by standard deviation and range when possible with the following formatting: (e.g., for age, 49 (16; 30–60), the mean is 49, the standard deviation is 16 and the range is 30–60. In the *Main Findings* column, effects related to EEG assessment at a specific time point are denoted by, e.g., “**T1:”** or “**T2:”**, and effects related to changes over time are denoted as “**T1 to T2:”**, or in a few cases “**T1 and T2”** or** “T1 to T3”**. Effects related to Pre vs Post treatment study effects, with usually at least T1 and T2 EEG, are denoted with **“Pre/Post:”**. In the *Time Window* column, the information provided is the last assessment time point of a study based on available study means or medians. Information that was unclear and/or not directly reported, and thus inferred, is denoted with *. Cells where information could not be determined froman article are denoted with **. ***List of Symbols and Acronyms:*** δ, delta (1–4 Hz); θ, theta (4–7 Hz); α, alpha (8–12 Hz); β, beta ( 13–30 or 13–40 Hz); γ, gamma (> 30 or 40 Hz); Active, Active group in a stimulation study; AUD, Alcohol Use Disorder; A-CBM, Alcohol-related memory activation-Cognitive Bias Modification; BEH, Behavioral; CAN, Cannabis Use Disorder; CAN-long, Cannabis use for 8 or more years; CAN-short, Cannabis use for less than 8 years; CBM, Cognitive Bias Modification; CUD, Cocaine Use Disorder; DTI, Distress Tolerance Intervention; EC, Eyes Closed; EO, Eyes Open; ERN, Error Related Negativity; ERP, Event Related Potential; GNG, Go/No-Go Task; Hf-rTMS, High Frequency Repetitive Transcranial Magnetic Stimulation; HVC, Health Video Control; iTBS, intermittent Theta Burst Stimulation; LONG, Longitudinal; LPP, Late Positive Potential; MBRP, Mindfulness-based relapse prevention; MID, Monetary Incentive Delay; MIX, Mixed Substance Use Disorders; MMN, Mismatch Negativity; MUD, Methamphetamine Use Disorder; Ne, Error Related Negativity (same as ERN); NIC, Tobacco Use Disorder; N-CBM, Neutral memory activation-Cognitive Bias Modification; OUD, Opioid Use Disorder; Pe, Error Related Positivity; rTMS, Repetitive Transcranial Magnetic Stimulation; s, seconds; Sham, Sham group or condition in a brain stimulation study; SST, Stop Signal Task; STIM, Stimulation; tDCS, Transcranial Direct Current Stimulation; T1, Earlier assessment time-point with EEG; T2, Later assessment time-point with or without EEG; T3 through T6, Other subsequent assessment time points.

### Quality and bias assessment

Two reviewers (AAK, RBS) assessed the quality and potential for bias of each study. Depending on the type of study being reviewed, the most generally appropriate rating tools were selected from the National Heart, Lung, and Blood Institute online resource. The selected Tool 1 was the *Quality Assessment Tool for Before-After (Pre-Post) Studies with No Control Group* (NIH, 2022b) with 11 questions for 29 studies. The selected Tool 2 was the *Quality Assessment of Controlled Intervention Studies* (NIH, 2022a) with 14 questions for 15 studies. Studies rated with Tool 1 consisted of longitudinal studies with at least two time points, and four pre-post studies. Studies rated with Tool 2 consisted of only pre-post studies reported as part of clinical trials. The questions for Tool 1 and 2 are listed in Supplementary Table 2 and Supplementary Table 3. Responses for each question were Yes (Y), No (N), Not Reported (NR), Not Applicable (NA), or could not be determined (CD). Based on responses and reviewer judgment, the process resulted in a rating of Good (G), Fair (F), or Poor (P) per study, and a percentage estimate of Yes responses for each study and question. Any disagreements were discussed and resolved by the two reviewers. However, in general, few studies provided detailed information required to fully respond to some of the Tool questions, and the best guess was used by reviewers when necessary. These quality ratings are included in Supplementary Table 4 and Supplementary Table 5. For question 7 (*Were the outcome measures prespecified, clearly defined, valid, reliable, and assessed consistently across all study participants?*) on Tool 1 outcomes were considered to be either EEG or outcome variables (i.e., abstinence) at T2. For question 10 (*Did the statistical methods examine changes in outcome measures from before to after the intervention? Were statistical tests done that provided p values for the pre-to-post changes?*) on Tool 1, the response was Yes only for longitudinal or pre-post treatment studies with EEG at T1 and T2. Questions 4 (*Were all eligible participants that met the prespecified entry criteria enrolled?*) and 11 (*Were outcome measures of interest taken multiple times before the intervention and multiple times after the intervention (i.e., did they use an interrupted time-series design)?*) on Tool 1 were generally deemed No or Not Reported across most studies rated with Tool 1.

In this review, T1 usually means the first (EEG) assessment, and T2 usually means the second assessment (of EEG and/or treatment-related outcomes), although a few studies had more than two time points. Our review of findings is selective, focusing mainly on the EEG findings at T1 that are associated with outcomes at T2 (and/or the longest follow-up). In the following section reviewing patterns reported across studies, ERP effects that are mentioned refer to amplitude effects unless otherwise specified (i.e., latency/delay). For negative-going components (e.g., ERN, N200), “increase” and “higher” refer to changes in the component. For example, increased ERN or higher ERN refers to increased negative amplitude in the ERN). For spectral effects, most were in terms of absolute power unless otherwise specified (i.e., relative or other). Effects relative to non-substance using controls are not mentioned in the narrative or tables.

## Results

### Characteristics of studies selected for review

Selected studies were heterogeneous, varying considerably in terms of the type of tasks used, sample characteristics and their reporting, length of abstinence at each assessed time point, and in specific EEG and analysis methods used. Types of SUDs covered across the studies included: 13 with AUD, seven with CUD, seven with MIX, six with NIC, four with CAN, four with MUD, and three with OUD.

**Figure 2:**
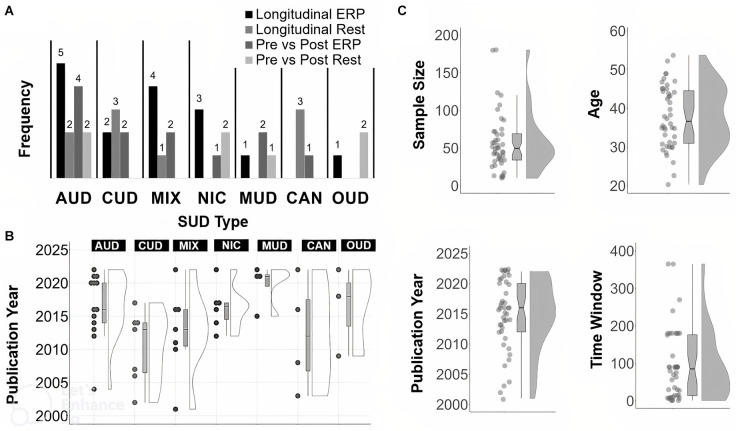
Summary of article features. Frequency count for each SUD type **(A)**, SUD type by year **(B)**, and, distribution and boxplot **(C)** of sample size, year, Time 1 to Time 2 in days, year of publication and age. For Time 2 assessments done the same day or session, the Time to Time 2 value for those studies was set to 1 day.

See Figure 2 for a summary of frequency counts (Figure 2A), year of publication per SUD type (Figure 2B), and year of publication, sample size, participant age, and time from T1 to T2 (Figure 2C). Among the included studies, 25 were longitudinal (i.e., T2 is usually greater than 1 week after T1). In terms of SUD type, the 25 longitudinal studies consisted of seven with AUD, five with CUD, three with NIC, five with MIX, three with CAN, one with OUD, and one with MUD. The other 19 studies were on pre- and post- brain stimulation (*n* = 12), pharmacological (*n* = 3) or behavioral (*n* = 3) treatments. A single study (*n* = 1) used both behavioral and stimulation treatments. In terms of SUD sample type, this set of 19 pre- and post- treatment articles consisted of six with AUD, two with CUD, three with NIC, three with MUD, two with OUD, two with MIX and one with CAN. Pre-post T2 follow-up assessments usually occurred the same session day or after multiple weeks at the end of treatment.

The mean sample size across all studies was 57.16 (SD: 38.33; median = 49.50; range: 10–180). The mean age across all studies was 37.75 years (SD: 8.02; median = 36.65; range: 20–54).The mean or median number of reported days between T1 and the last time-point (usually T2) across all studies was 76.26 days (SD: 76.48; median = 62.50; range: 1–365). The mean percentage of males across studies was 71.02% (SD: 19.22; median = 71; range: 31–100).

Other study details mainly associated with quality and reproducibility included the following. Bio-verification of abstinence (i.e., toxicology screens) was reported in 21% of studies at T1 only and 37% of studies at T1 and T2. A non-SUD control group was used in 40% of studies (usually at T1). Education details were reported in 57% of studies. Ethnicity details were reported in 32% of studies. Some limitations across studies were sparsity in terms of the number of studies per type of task and per SUD, relatively small sample sizes, most T1 to T2 periods being brief and less than 6 months, and lack of control for various confounding factors such as polysubstance use. Further, multiple studies had issues with unbalanced sex ratios, drop-out or attrition, or incomplete reporting of EEG processing details.

Studies were published from the following countries, based on first author’s affiliation: United States (*n* = 16), China (*n* = 5), Brazil (*n* = 4), Belgium (*n* = 3), Austria (*n* = 2), Iran (*n* = 2), Netherlands (*n* = 2), Spain (*n* = 2), Australia (*n* = 1), Germany (*n* = 1), Ireland (*n* = 1), Israel (*n* = 1), Italy (*n* = 1), Malaysia (*n* = 1), Poland (*n* = 1), and Switzerland (*n* = 1).

Tasks used in ERP studies varied in methodological details (recording length, number of trials, type of stimuli) but primarily used established protocols or their variants that target particular neurocognitive processes, including P3-generating visual oddball tasks (indexing attention/executive function, working memory updating), P3/ERN/N2-generating Go/No-Go tasks (indexing response inhibition, performance monitoring), and P3/LPP-generating affective/drug image viewing, also known as cue-reactivity tasks (indexing motivational salience). Most studies examining EEG spectra and resting-state (spontaneous) oscillations (e.g., beta) used eyes-closed recordings, and two used eyes-open recordings. The mean duration of recording times in spectra studies was 298.35 s (SD: 261.10 s median = 180; range 32–1,200 s). The mean number of EEG channels across studies was 48.27 (SD: 34.40; median = 32; range: 3–128) with most analyses focused on a small subset of channels (<10) specific to the ERP analyses of interest, or a small number of regional averages (e.g., frontal, central, and posterior channels). Source estimation was reported in 22% of studies. Some form of EEG-derived classification accuracy was reported in 25% of studies, mainly in those that examined abstinence as a binary outcome at a follow-up.

### Studies of abstinence and treatment-as-usual outcomes

#### Event-related potentials (ERPs)

Among 16 ERP studies, five were in AUD, two in CUD, three in NIC, one in OUD, one in MUD and four in MIX. ERP predictors of abstinence status and other treatment outcomes from these studies are reported here. Most studies used abstinence vs. relapse as a binary outcome variable and thus we report “abstinence” to mean abstinence/relapse as one binary factor unless otherwise specified. Longitudinal periods are estimated from means or medians reported in each study, with details listed in Table 1 and Supplementary Table 1.

##### AUD

One report showed that lower visual-oddball P3 at T1 predicted continued abstinence at 3 months follow-up (T2; Petit et al., 2015). The study further showed that the P3, from inferior-medial temporal sources and elicited by alcohol (relative to non-alcohol) cues, added utility in predicting relapse above and beyond the family history of alcoholism, craving, and depression (Petit et al., 2015). Another study reported higher alcohol No-Go vs. Go P3 at T1 significantly predicted, along with impulsivity and the likelihood of abstinence at 3 months and even after accounting for the effects of age, craving, and family history of alcohol use (Petit et al., 2014). However, another study reported that P3 elicited by a Go/No-Go task did not significantly differentiate between abstainers and relapsers (Batschelet et al., 2021), although a trend of higher alcohol vs. neutral No-Go P3 was observed at T1 in only the individuals who relapsed at 3 months follow-up (T2; Batschelet et al., 2021). The same study also reported higher alcohol vs. neutral No-Go frontal N2, with anterior cingulate, medial-frontal, inferior parietal, and temporal sources, was associated with higher craving at T2 (Batschelet et al., 2021). Another study using a Go/No-Go task with alcohol and non-alcohol neutral cues reported that higher alcohol No-Go N170 at T1 discriminated between abstainers and relapsers at 3 months follow-up (T2; Matheus-Roth et al., 2016). In addition, the study indicated faster P100 latency at T1 was associated with higher depressive symptoms at T2 (Matheus-Roth et al., 2016). Lastly, a study showed that a longitudinal increase in alcohol No-Go vs. Go P3 from T1 to T2 (~17 days post-detox) predicted abstinence at 3 months (Campanella et al., 2020). The study also reported no longitudinal change in P3 elicited by a visual oddball alcohol task from T1 and T2 (Campanella et al., 2020).

##### CUD

In one study, lower Eriksen flanker ERN at T1 predicted greater last month cocaine use, craving, and cocaine use severity at 3 months (T2; Marhe et al., 2013). The ERN added utility in predicting recent cocaine use above and beyond clinical variables (cocaine craving and use severity; Marhe et al., 2013). A study in treatment-seeking CUD showed a longitudinal increase in LPP elicited by pleasant relative to drug cues from T1 to T2 (over ~6 months of abstinence or reduced cocaine use), and this relative LPP increase was associated with lower craving at T2 (Parvaz et al., 2017).

##### NIC

One study showed that higher LPP elicited by pleasant cues at T1 predicted abstinence at T2 (10 and 24-week follow-ups; Versace et al., 2012). Another study showed lower Go/No-Go P3 and higher N2 at T1 predicted relapse (Luijten et al., 2016). The same study also showed trends for associations of smaller Pe with relapse as well as lower ERN with smoking behavior. A third report, using a stop-signal task, indicated faster left fronto-central N2 latency, higher fronto-lateral P3, and lower left central and right frontal P3 elicited by correct stop trials at T1 (pre-quit baseline) predicted longer time to relapse over a year (Lespine et al., 2022). The same study also noted increased right central P3 (failed stop trials) and decreased frontal and left posterior late P3 (correct stop trials) from T1 to T3 (4 weeks) that predicted longer time to relapse (Lespine et al., 2022). However, the study reported that the EEG metrics were weak predictors of time to relapse relative to psychological and affective state variables (Lespine et al., 2022).

##### MUD

An ERP study reported abstinence-related decreases in left anterior P3 to methamphetamine words at 3 and 6 months, along with correlations of decreases in P3 with decreases in craving over abstinence (Haifeng et al., 2015).

##### OUD

An ERP study showed that less attenuation of the startle-elicited pleasant vs. drug cue P3 at T1 predicted recent weekly heroin use at 6 months (T2), though this ERP metric was a weak predictor of recent heroin use relative to subjective picture ratings (Lubman et al., 2009).

##### MIX

Lower auditory-oddball midline P3a at T1 was found in treatment non-completers at ~2 months (T2; Anderson et al., 2011). Using stepwise regression analysis, the study also reported that P3a was the only predictor of treatment outcome relative to age, education, severity, and IQ (Anderson et al., 2011). In another study, lower visual oddball P3 at T1 predicted treatment non-completion at 6 months, and midline parietal target P3 outperformed non-EEG variables in predicting treatment completion (Wan et al., 2010). In two studies on similar samples, the first report indicated lower Go/No-Go P2 and ERN, and higher Pe at T1. Steele et al. (2014) predicted treatment non-completion, with P2 and Pe being the stronger predictors relative to non-EEG variables. The second report indicated lower visual oddball P3a at T1 and higher visual distractor N2 predicted treatment non-completion at T2 (Fink et al., 2016).

### Interim summary: ERP

The evidence for ERPs in association with abstinence and treatment outcomes is most developed in AUD. Although heterogeneous, the evidence base from other SUD studies is broadly consistent with findings from AUD, in that ERPs have strong potential as prognostic and prospective biomarkers of treatment-related outcomes. Overall, higher cognitive (e.g., visual oddball) P3, lower affective (cue-elicited) P3 (or LPP), and higher (i.e., more negative) N2 and ERN at T1 were predictors of abstinence and positive treatment outcomes at T2. In terms of longitudinal changes from T1 to T2, an increase in cognitive P3, a decrease in affective P3 (or LPP), and an increase in response to drug-unrelated positive stimuli were associated with longer abstinence or positive outcomes.

Importantly, many of these studies used SUD-specific stimuli (i.e., drug-related cues to assess cue-reactivity or to create a drug-related context), and cognitive control (mainly Go/No-Go) tasks, which are critical in examining dysregulation of motivational and inhibitory control processes, respectively, in experimental SUD research. In some studies, the oddball and Go/No-Go effects were elicited without SUD-specific stimuli, and thus reflect the engagement of more general cognitive factors (e.g., working memory updating, inhibitory control). There is also a small but consistent evidence base for greater LPP (or P3) in response to pleasant (relative to drug) stimuli at T1, or its increase from T1 to T2, being associated with better outcomes (i.e., longer abstinence and lower craving). However, we also note that some studies reported no relapse-related P3 effects (Petit et al., 2015; Batschelet et al., 2021) or no longitudinal abstinence-mediated changes in LPP from T1 to T2 (Parvaz et al., 2017; Campanella et al., 2020).

#### Spectral studies of abstinence

Among nine studies that examined EEG oscillations in SUD samples, two were in AUD, three in CUD, three in CAN, and one in MIX.

##### AUD

One study reported that lower frontal-parietal beta, higher frontal delta, and higher global relative delta and theta power at T1 predicted abstinence at 6 months (T2; Saletu-Zyhlarz et al., 2004). In contrast, the same study reported higher frontal beta2 (20–25 Hz) and global relative beta, along with lower frontal delta, theta, and frontal-occipital alpha at T1 predicted relapse at T2 (Saletu-Zyhlarz et al., 2004). Another study found that higher delta connectivity between dorsolateral prefrontal and inferior parietal regions, and between anterior insula and inferior frontal junction regions, at T1 predicted abstinence at 12 months (T2; Januszko et al., 2021).

##### CUD

One study examined EEG subtypes and reported that higher centro-parietal beta (12.5–25 Hz) and higher alpha at T1 predicted abstinence at 25 weeks (T2; Prichep et al., 2002). Another study found higher left temporal beta1 (14–25 Hz) and right temporal delta, as well as higher beta2 (25–40 Hz), at T1 predicted abstinence at 1 month (T2; Levin et al., 2007). The same study also indicated trends due to abstinence including increased right central and occipital delta, and decreased left frontal spectra from delta to beta (Levin et al., 2007). Another study reported that higher cordance, a measure of agreement between absolute and relative power, at T1 predicted treatment completion at 2 months (T2; Venneman et al., 2006). The study also noted that EEG metrics were better predictors of treatment outcomes than clinical variables such as depression and craving (Venneman et al., 2006).

##### CAN

One study found no EEG changes with abstinence from T1 to T2 (2 weeks) but noted trends for associations between older age and later onset of first use with beta T1 to T2 increases (Allsop and Copeland, 2016). Another study reported lower theta and alpha at both T1 and 1 month (T2; Herning et al., 2003). Another study also found no EEG abstinence effects in individuals with longer (>8 years) vs. shorter histories of cannabis, though long-term users evinced lower occipital alpha peak frequency as well as lower posterior high alpha and beta2 (25–40 Hz) at both T1 and 1 month (T2; Herning et al., 2008).

##### MIX

One study showed that higher frontal beta2 (20–40 Hz) at T1 predicted relapse at 6 months (T2) more accurately than did other variables such as substance use, family history, anxiety, and conduct disorder (Bauer, 2001b).

### Interim summary: spectra

Overall, although the current evidence is heterogeneous and sparse across most SUDs, there is a consistent evidence for lower beta as a SUD-general (mostly AUD) resting EEG biomarker of abstinence (Allsop and Copeland, 2016) and positive treatment outcomes. In addition to lower beta power, the reviewed patterns also suggest higher power in low frequency bands (delta and theta) predict abstinence in AUD and MIX but predict relapse in CUD. Some studies also note changes in theta and alpha bands in relation to abstinence and relapse but these effects and their association with clinical outcomes need further replication.

### Treatment studies

#### Brain stimulation

There were 12 EEG/ERP studies with brain stimulation treatment: four in AUD, two in CUD, three in MUD, one in NIC, one in OUD and one in MIX. Of these, six studies examined ERP metrics (two in AUD, two in CUD, one in MUD and one in MIX), four studies examined spectral EEG metrics (one in AUD, one in MUD, one in OUD, and one in NIC). Further, one study in MUD examined both ERP and spectral EEG metrics, and one study in AUD examined graph-theoretical metrics using stimulation-evoked cortical inhibition. Of these, nine studies used the left dorsolateral prefrontal cortex (dlPFC) as the stimulation target, two studies used bilateral dlPFC targets, and one study used a right inferior frontal target. In terms of stimulation methods, eight studies used transcranial Direct Current Stimulation (tDCS), three studies used repetitive Transcranial Magnetic Stimulation (rTMS), and one study used intermittent theta burst stimulation (iTBS). All effects reported below are in relation to pre- and post-stimulation treatment vs. sham, unless otherwise noted.

##### AUD

One study examined the effects of 5 weekly sessions with 20-min tDCS (2 mA, left dlPFC) and found decreased alcohol and neutral cue LPP with frontal sources (da Silva et al., 2013). The study also indicated stimulation-related decreases in craving and depression, no effects in terms of abstinence, and trends to increased relapse in the active stimulation condition (da Silva et al., 2013). Another study examined the effects of a single session with 10-min tDCS (1 mA, left dlPFC) and found increased frontal auditory alcohol-cue P3 and decreased centro-parietal P3 for the Lesch IV AUD-subtype group (i.e., those in whom, according to Lesch Typology (Schlaff et al., 2011), drinking is caused by compulsion and early onset of severe somatic disease), with opposite patterns in the Lesch II group (i.e., those in whom, according to Lesch Typology, drinking is primarily used to diminish anxiety; Nakamura-Palacios et al., 2012). The same study indicated stimulation-related increases in frontal-executive assessment scores (Nakamura-Palacios et al., 2012). Another study examined the effects of one session with ~30 min TMS (inhibitory paired-pulse paradigm, 75 paired-pulses, 1 s interstimulus interval, bilateral dlPFC) with the left dlPFC target condition leading to increased clustering coefficient (i.e., connectivity of nearby nodes) and decreased efficiency (i.e., shortest path length reflecting integrated processing). The same study also indicated links between decreased mean degree (number of in and out connections, hubness) and clustering coefficient to greater severity of past use (Naim-Feil et al., 2022).

One spectral study examined the effects of four sessions with ~5 min high frequency rTMS (10 Hz, left dlPFC) across 2 weeks and found increased delta relative to gamma (Del Felice et al., 2016). The study also reported, in the active group only, positive correlations of central alpha and theta with depression/somatization (T1) as well as negative correlations of alpha2 and beta with Stroop performance (Del Felice et al., 2016).

##### CUD

One study examined the effects of one session with 20-min tDCS (2 mA, left dlPFC) and found decreased crack-cue N2 with anterior cingulate sources (Conti and Nakamura-Palacios, 2014). A second study with a similar sample and using dlPFC P3 estimates, found single-session tDCS led to left P3 increases to neutral cues and decreases to crack cues, as well as trends suggesting decreased right P3 to crack cues (Conti et al., 2014). The study also indicated that five tDCS sessions over 10 days led to increased crack-cue reactivity at frontopolar, orbitofrontal, dorsolateral prefrontal, and anterior cingulate regions (Conti et al., 2014).

##### NIC

One spectral study examined the effects of one 12-min session of high frequency rTMS (10 Hz, left dlPFC) and found increased gamma, decreased delta and alpha, and decreased craving (Pripfl et al., 2014).

##### MUD

One study examined the effects of 20 sessions with 5-min iTBS (theta, 3-pulse 50 Hz bursts, left dlPFC) over 4 weeks and found no changes in methamphetamine vs. neutral cue ERPs but indicated decreases in frontal-central beta (Chen et al., 2021). A second study with one session of 20-min tDCS (2 mA, left dlPFC) reported stimulation-related decreased drug vs. neutral P3 (at post-stimulation only) that was correlated with decreased resting-state graph-theoretical (i.e., network) efficiency in multiple areas including bilateral medial temporal, left superior parietal and angular gyrus, and right frontal and supplementary motor regions (Khajehpour et al., 2022). A spectral study examining the effects of 20 sessions with 4-min iTBS (theta, 3-pulse 50 Hz, left dlPFC) over 10 days found decreased left prefrontal delta that was linked to treatment-related increases in working memory performance (Zhang et al., 2021a).

##### OUD

One spectral study examined the effects of 10 sessions of 20-min tDCS (2 mA, left/right dlPFC) over 10 days (Mostafavi et al., 2022). Left anode/right cathode stimulation decreased central and bilateral frontal delta, bilateral frontal and right occipital-parietal beta2 (25–30 Hz), as well as increased connectivity at right fronto-central and left fronto-temporal delta, fronto-central and right fronto-temporal beta, and parieto-temporal alpha (Mostafavi et al., 2022). Right anode/left cathode stimulation decreased left occipital alpha and beta1 (18–25 Hz), as well as increased connectivity at left frontal-parietal theta and alpha, and right centro-frontal delta (Mostafavi et al., 2022).

##### MIX

One study examined the effects of five sessions of 20-min tDCS (2 mA, left dlPFC cathode, right anode) over 5–10 days and found increased drug vs. neutral cue P3 with ventromedial prefrontal sources from T1 to T2 (Nakamura-Palacios et al., 2016). The same study indicated that these ventromedial P3 increases predicted abstinence during and after treatment (Nakamura-Palacios et al., 2016).

### Interim summary: brain stimulation

The literature on the effects of brain stimulation in SUD is still preliminary, with small samples and no longitudinal follow-ups, sparse and heterogeneous findings, and several studies showing no clear EEG-specific pre vs. post stimulation effects. Across the reviewed studies, the evidence suggests that stimulation techniques can lead to a change in ERPs and in spectra, alongside beneficial changes in clinical variables related to treatment outcomes, such as craving and cognitive function. In particular, the limited evidence is partly congruent with treatment expectations based on other SUD-EEG work, e.g., decreased LPP and N2 drug-cue reactivity, increased delta, decreased beta, and suggests that ERPs in SUD samples can be modulated by stimulation effects. However, the current evidence base for the directionality of stimulation effects on ERP amplitude and spectral power metrics remains sparse and mixed. At best, the studies reviewed here suggest the feasibility and potential utility of brain stimulation methods for modifying treatment-relevant electrophysiological brain activity in SUD treatment research.

#### Pharmacological and behavioral treatments

These included seven studies: three pharmacological (one in OUD, one in NIC, one in MIX), three behavioral (one in AUD, one in NIC, one in CAN), and one in AUD with both behavioral and brain stimulation (tDCS, right inferior gyrus) treatments.

##### AUD

One study examined the effects of alcohol-related memory activation and cognitive-bias modification (A+CBM) with four 20 min sessions over 1 week. The study found that A+CBM was associated with increased alpha connectivity and greater avoidance of alcohol-related cues from T1 to T2. The same study also reported higher central beta at T2 for the A+CBM group relative to other treatment groups (Martinez-Maldonado et al., 2020). A second study examined the effects of combined treatment, with eight 2-h sessions over 2–3 months, including 90 min of mindfulness-based relapse prevention (i.e., meditation) and 30 min of tDCS (2 mA, right inferior frontal gyrus) per session (Brown et al., 2020). The report indicated that whereas mindfulness led to decreased alcohol-cue LPP, brain stimulation led to increased alcohol-cue LPP, and that attending more mindfulness sessions was linked to decreased craving (Brown et al., 2020).

##### NIC

One study examined the effects of bupropion and varenicline over 12 weeks and found that those participants with greater pleasant relative to cigarette cue LPP at T1 had higher overall abstinence relative to placebo at the end of treatment, and at 3 and 6 month follow-ups (Cinciripini et al., 2017). Abstinence rates were higher (doubly so) for varenicline than bupropion (Cinciripini et al., 2017). A study examining the effects of a hypnosis disgust-suggestion intervention (30 min) found increased delta along with decreased alpha and beta (Li et al., 2017). The same study also reported that T1 to T2 increases in delta connectivity were correlated with decreases in craving (Li et al., 2017).

##### CAN

A study examining the effects of a distress-tolerance intervention (two sessions, one per week, 30 min psychoeducation, 30 min idiographic distress exposure) found no changes in cannabis and threat cue LPPs (Macatee et al., 2022). However, the study noted links between higher post-stressor cannabis cue LPP and greater severity/chronicity at 4-month followup (Macatee et al., 2022).

##### OUD

One study examined the effects of methadone maintenance treatment over 10 weeks in individuals with heroin use disorder and found the treatment was associated with delayed auditory oddball P3 (Motlagh et al., 2018). The report also noted T1 to T2 trends for decreased frontal beta, increased temporal delta and alpha, as well as positive correlations of theta with subjective withdrawal (Motlagh et al., 2018).

##### MIX

One study examined the effects of piramate treatment over 5 weeks and found that the higher dosage condition had decreased in both affective and drug cue LPPs at T2 (Robinson et al., 2022). The report also indicated that the LPP effects did not mediate the change in clinical variables (e.g., post-quit use, craving; Robinson et al., 2022).

### Interim summary: pharmacological and behavioral

Overall, the current set of behavioral and pharmacological studies is small and heterogeneous, with some studies reporting lack of clear treatment effects (Martinez-Maldonado et al., 2020; Macatee et al., 2022), lack of mediation of clinical outcome variables by EEG metrics (Robinson et al., 2022), or no links between EEG metrics and clinical variables (Brown et al., 2020). Nevertheless, results showing decreases in higher frequency bands and increases in lower frequency bands with treatment, as well as higher LPP to pleasant cues predicting positive outcomes, are generally in agreement with the EEG-SUD literature reviewed above in previous sections.

### Quality and bias assessment

In ratings using NIH tools, most of the studies included were of good (81%, Total *N* = 36, 23 in Tool 1, 13 in Tool 2) or fair quality (18%, Total *N* = 8, six in Tool 1, two in Tool 2), with no studies scored as poor quality. The percentage of studies with Yes ratings across all questions, averaged across studies, was 69% (SD: 11.06) for Tool 1 (56%, SD: 9.05, when discounting Questions four and 11) and 66.67% (SD: 13.47) for Tool 2. For Tool 1, questions with zero percent Yes ratings included Question four (*Were all eligible participants that met the prespecified entry criteria enrolled?*) and Question 11 [*Were outcome measures of interest taken multiple times before the intervention and multiple times after the intervention (i.e., did they use an interrupted time-series design)?*]. The next questions with low percentages of Yes responses from Tool 1 included Question eight (3%, *Were the people assessing the outcomes blinded to the participants’ exposures/interventions?*), Question five (31%, *Was the sample size sufficiently large to provide confidence in the findings?*), and Question 10 (34%, *Did the statistical methods examine changes in outcome measures from before to after the intervention? Were statistical tests done that provided p values for the pre-to-post changes?*). Questions with zero or low percentages of Yes responses from Tool 2 included Question 14 (0%, *Were all randomized participants analyzed in the group to which they were originally assigned, i.e., did they use an intention-to-treat analysis?*), Question 12 (13%, *Did the authors report that the sample size was sufficiently large to be able to detect a difference in the main outcome between groups with at least 80% power?*), Question four (20%, *Were study participants and providers blinded to treatment group assignment?*), Question 14 (20%, *Were the people assessing the outcomes blinded to the participants’ group assignments?*). Although multiple studies considered with Tool 2 mentioned being part of a clinical trial or that group assignments were randomized, few provided extended specific details on blinding across participants, procedures and study staff.

### General discussion

The rationale for this scoping review was to examine cumulative knowledge since the year 2000 regarding EEG markers related to SUD treatment changes and outcomes. We examined potential EEG biomarkers in longitudinal and multi time-point studies that assessed within-subject changes following abstinence or treatment in EEG brain function, or that associated EEG at an earlier time point to clinical assessments at a later time-point. These studies are useful for identifying EEG patterns at T1 and potential trajectories of EEG changes after T1 associated with positive treatment outcomes and recovery in individuals with SUD. Next, we overview some main patterns and issues, with a general caveat that interpretations are limited considering the heterogeneity of findings, methods, and samples across the reviewed studies.

Overall, the reviewed research suggests that there are ERP (mainly oddball P3, possibly No-Go N2 and cue-elicited LPP) and spectral (mainly beta, possibly delta, alpha and theta) changes in EEG associated with abstinence and treatment. We note there is relatively good and consistent evidence to date for higher cognitive P3 and lower beta at T1 as predictors of abstinence and positive treatment outcomes at T2, and good evidence for normalization of EEG (e.g., increased cognitive P3 and decreased beta) at T2. Longitudinally (i.e., from T1 to T2) increase in cognitive (e.g., visual oddball) P3, and decrease in affective (drug cue-elicited) P3 (or LPP), were associated with longer abstinence or positive outcomes. Higher LPPs elicited by pleasant stimuli at T1, and their increase from T1 to T2, were also related to better outcomes. These results are consistent with SUD models of higher incentive salience of drugs in individuals with chronic substance use (Lüscher et al., 2020). Although there is less data on N2 relative to the P3, higher (i.e., more negative) No-Go N2 and ERN (reflecting better inhibitory control) seem to predict better outcomes, which together with the P3/LPP results, are in line with the Impaired Response Inhibition and Salience Attribution (iRISA) model of SUD (Zilverstand and Goldstein, 2020; Ceceli et al., 2022). However, future SUD-related cognitive and affective P3 research studies need to better contextualize their models and findings with the extensive current knowledge about P3, and ERPs more generally, such as the distributed network of P3 cortical (midfrontal and frontal-parietal) and deep-brain generators (Pfabigan et al., 2014; Ragazzoni et al., 2019; Li et al., 2020; de la Salle et al., 2021) and EEG/ERP changes indirectly related to SUD treatment, such as those due to intoxication (Fairbairn et al., 2021), binge-drinking (Lees et al., 2019) and ageing (van Dinteren et al., 2014). Results from studies examining EEG spectra suggest that higher power in low frequency bands (delta and theta) and lower power in high frequency bands (beta) predict better clinical outcomes. Relatedly, parietal delta and frontal theta oscillations are also known to be involved with the generation and modulation of the P3 (Başar-Eroglu et al., 1992; Bachman and Bernat, 2018), and therefore, increases in P3 and in low frequency EEG activity may reflect changes in similar neural substrates.

The evidence for the involvement of beta is also consistent with previous SUD-EEG reviews (Liu et al., 2022), including patterns suggesting that increase and decrease in beta are associated with worse and better outcomes, respectively. There is also accumulating evidence in this and past reviews for delta, theta, and alpha effects related to treatment outcomes that requires replication and extension. Beta (in particular, central beta, variously reported from >13 to <40 Hz) has established roles in somatosensory and motor processes (Neuper and Pfurtscheller, 2001; Jenkinson and Brown, 2011; Pavlidou et al., 2014; Khanna and Carmena, 2015; Little et al., 2019; Barone and Rossiter, 2021). Considerable research also points to its involvement in executive control, working memory, and brain network integration (Betti et al., 2021). In particular, beta reflects a top-down “status quo” signal maintaining endogenous rules or long-term “priors” regarding environmental stimuli and behaviors involved with binding of large scale sensorimotor networks (Engel and Fries, 2010; Spitzer and Haegens, 2017; Betti et al., 2021; Michail et al., 2022). There is also strong evidence for beta’s role in the genetic risk for AUD (Porjesz et al., 2002; Smit et al., 2018), binge drinking (Lees et al., 2019; Almeida-Antunes et al., 2022), and also in drug craving, e.g., in AUD (Huang et al., 2018) and MUD (Zhao D. et al., 2021), an important factor related to relapse (Serre et al., 2018; Stohs et al., 2019). Overall, dysregulated beta in SUD and its modification by abstinence or treatment, and its association with a range of neurocognitive processes, suggests that higher beta may serve as a marker of neurobehavioral deficits in motor and executive control, impulsivity, and endogenously-driven habits in SUD. Importantly, from the included studies it appears that these impairments may have been *caused* by substance use, as evident by reductions in beta with abstinence and treatment suggesting recovery of these SUD-related deficits.

We found partial evidence for EEG or ERP “recovery” with abstinence and treatment, which is in line with past ERP (Campanella et al., 2014; Zhang et al., 2021b) and EEG (Liu et al., 2022) reviews. In particular, past reviews highlighted existing research that suggests recovery effects in oddball P3 (Campanella et al., 2014; Zhang et al., 2021b) or resting EEG spectra for beta with abstinence in AUD (Liu et al., 2022), as well as initial evidence for abstinence-related increases in low-frequency bands (e.g., delta, theta) relative to decreases in higher-frequency bands (Liu et al., 2022). The patterns of results that have emerged from this review are generally concordant with those from existing longitudinal structural, functional, and neurochemical neuroimaging studies of sustained abstinence that also show evidence of partial recovery (normalization), especially across fronto-parietal, insula, and other brain regions associated with SUD (Stewart et al., 2019b; Parvaz et al., 2022). However, as in SUD-EEG research, the neuroimaging evidence base is also challenged in terms of heterogeneity, sparsity across SUDs, and lack of longitudinal studies. With continued systematic validation and reliability work on SUD-EEG, and coordination with multi-modal imaging findings, EEG metrics may serve as potential biomarkers that can be targeted for routine use in SUD treatments.

For pre-post treatment studies we found proof-of-concept initial evidence for the impact of brain stimulation on SUD-related ERP and EEG metrics, and scarce but promising initial evidence for changes in EEG with pharmacological and behavioral treatments. Brain stimulation studies are particularly exciting, and have the potential to target neurobiological mechanisms that underpin SUD and related behaviors (Hauer et al., 2019; Wischnewski et al., 2019; Zhang et al., 2019; Habelt et al., 2020; Bollen et al., 2022). Surprisingly, although pharmacological and behavioral interventions have been examined in SUDs for a few decades, only a few studies have used EEG biomarkers to study SUD treatment outcomes. Overall, however, results from treatment studies are largely consistent with those from abstinence studies showing an increase in P3 and decrease in beta frequency related to better treatment outcomes. Nevertheless, results from pharmacological and behavioral treatment studies were more heterogeneous than those from brain stimulation studies. Indeed, integration and extension of this literature is required in relation to the broader evidence base regarding short term and longitudinal drug treatment effects on EEG metrics, as well as various forms and active ingredients of extant effective treatments.

One major challenge to current knowledge regarding SUD-EEG biomarkers is that dysregulated ERPs and EEG are not unique to SUD and suggest a need for transdiagnostic frame works. For example, lower P3 is also seen in other disorders, i.e., depression (Bruder et al., 2012; Klawohn et al., 2020), schizophrenia (Qiu et al., 2014), bipolar (Wada et al., 2019), attention deficit/hyper-activity (Kaiser et al., 2020), autism (Cui et al., 2017), psychopathy (Gao and Raine, 2009), panic (Howe et al., 2014), and Alzheimer’s (Hedges et al., 2016), suggesting that P3 may be a trans-diagnostic factor (i.e., not specific to SUD) related to deficits in P3-associated attentional, working memory, and behavioral updating processes. Similarly, there is evidence for beta as a transdiagnostic rather than SUD-specific factor, as suggested by higher beta in insomnia (Zhao W. et al., 2021), psychosocial stress (Vanhollebeke et al., 2022), and Parkinson’s (Cagnan et al., 2019), and dysregulated (or decreased) beta in externalizing disorder (Rudo-Hutt, 2015), traumatic brain injury (Allen et al., 2021) and epilepsy (Dharan et al., 2021). In addition, theta deserves more research attention, as it is (like beta) a heritable marker of AUD (Meyers et al., 2021), involved with working memory and executive control (Cavanagh and Frank, 2014), and a well-studied biomarker in depression (Jamieson et al., 2022) and schizophrenia (Hirano and Uhlhaas, 2021). Overall, these trans-diagnostic issues also reflect the known high comorbidity of SUD and psychiatric disorders, which suggests a great potential for future synergy by combining SUD and psychiatric (e.g., depression) treatment efforts using EEG. However, we note that we formally excluded any studies focused mainly on comorbid psychiatric disorders (e.g., mood and anxiety disorders), in order to be consistent with past reviews and with most of the included studies wherein comorbid diagnoses were exclusionary. Nevertheless, given the considerable prevalence of psychiatric comorbidity with SUD, adequately powered studies should be conducted that can tease apart the independent and interactive effects of these comorbidities with SUD. The non-specificity of SUD-related findings in relation to many other disorders may also be due to the use of more “general” rather than disorder-specific EEG metrics and tasks. Encouragingly however, many studies reviewed used SUD-specific stimuli (e.g., cue-reactivity tasks) to better capture SUD-specific processes. Further, as we noted in interim summaries, though some studies reported that EEG metrics were strong predictors of outcomes at follow-up, other studies noted null effects in terms of EEG metrics or pre-post treatment EEG changes.

One major caveat of the current evidence base is that more remains to be known especially about individuals with SUD that do not seek treatment and/or do not participate in research studies, and those who drop out of treatment or treatment studies (Biele et al., 2019). Thus, SUD treatment-seekers may be a self-selected group, perhaps with less neurobiological vulnerability and/or be more apt to neural recovery compared to those who do not seek treatment, or are unable to quit or maintain abstinence (e.g., non-treatment-seekers and/or relapsers). Alternatively, as many individuals successfully recover from SUDs on their own without ever seeking treatment (i.e., spontaneous recovery), treatment-seeking individuals may represent more chronic and severe cases. Regardless of their SUD severity phenotype, treatment-seeking individuals with SUD are underrepresented in current research and deserve more attention. In addition, better accounting of the influence of potential confounders (e.g., age, polysubstance use, baseline SUD severity) is warranted in these studies. Lastly, there is a predominance of studies examining treatment-related changes in individuals with AUD, whereas treatment, abstinence, and recovery in other SUDs (e.g., opiates, methamphetamine, cannabis) are understudied, representing a critical gap in this area of research relative to current population trends. Nevertheless, the literature is growing, and we anticipate that future studies with larger sample sizes and longer follow-up periods will help clarify these issues.

A general goal of SUD brain research is to leverage data to improve prognostic outcomes in individuals with SUDs. Thus, linking EEG brain changes to clinical improvements is fundamental, and the current review suggests that EEG metrics do have associations with clinical outcome variables such as craving, and in some cases out perform clinical variables in predicting abstinence. However, the full breadth of improved clinical outcomes and their relation to longitudinal neural changes over abstinence and post-treatment remains to be discerned by future research.

### Limitations of the current review

Multiple factors limit the generalizability of the current findings. We did not include several interesting and related studies that did not fit our selection criteria. These included an estimated ~20 studies that were: (1) published before 2000 (~5 studies); (2) focused on sleep or neurofeedback (~7 studies); (3) used short-term and/or long-term abstainer samples (~5 studies); (4) used SUD samples that did not receive treatment or abstain (~3 studies). Further, we did not integrate the review findings with current knowledge regarding: (5) intoxication, binge-drinking, and chronic substance use effects on EEG/ERPs; (6) non-EEG treatment outcome predictors such as high impulsivity and low inhibitory control, concepts with an extensive behavioral and brain imaging literature (Vassileva and Conrod, 2019; Lees et al., 2021; Lutz et al., 2021); (7) fMRI-based functional, structural, and connectivity models of SUD treatment changes (Stewart et al., 2019b; Parvaz et al., 2022); and (8) SUD subtypes (Schwartz et al., 2010; Blonigen et al., 2016; Müller et al., 2020) or trajectories (Haller et al., 2010; McCabe et al., 2019; Infante et al., 2020). Moreover, (9) only a dozen of the reviewed studies were from non-Western countries, reflecting a Western bias limiting the generalizability of findings; and (10) the time frames in brain stimulation studies were usually quite brief (i.e., pre- and post- EEG on the same day), and although informative for neuromodulation-mediated changes in brain function, have limited utility in predicting longer-term treatment-related changes or relapse.

### Recommendations

This section integrates most limitations and recommendations gathered from past reviews (Bauer, 2001a; Kouri and Lukas, 2001; Campanella et al., 2014, 2019; Houston and Schlienz, 2018; Hamidovic and Wang, 2019; Stewart et al., 2019b; Jurado-Barba et al., 2020; Zhang et al., 2021b; Liu et al., 2022) and from this current review, focusing on how limitations of existing studies can be minimized, suggesting paths to enhance the validity and usefulness of SUD-EEG studies, and outlining possible future directions for research.

Suggested **basic methodological enhancements** include increasing consistent and complete reporting of participant demographics and backgrounds, polysubstance use assessment, and EEG processing details, as well as regular use of toxicology screens to confirm longitudinal abstinence and samples with balanced sex ratios. One of the most important needs, particularly in longitudinal SUD abstinence and/or treatment studies, is **larger sample sizes** (i.e., multi-center longitudinal or prospective cohort studies with *N* > 1,000, such as ABCD (Karcher and Barch, 2021) with fMRI or ENIGMA (Smit et al., 2021) with EEG, for reproducible (Pernet et al., 2020) brain function studies (Marek et al., 2022) to have adequate statistical power (Grady et al., 2021) and capture smaller nuanced effects that may otherwise get masked, which is especially required for complex multi-factorial diseases such as SUD. **Broadening treatment outcome targets** would allow studies to go beyond binary outcomes (i.e., abstainers vs. relapsers, treatment completion vs. non-completion, although craving and severity are often assessed as well). However, a wide array of post-treatment outcomes may be assessed including harm-minimization, quality of life, relapse episodes, social capital, insight, stress, exercise, sleep, and a range of other factors (Donovan et al., 2012; Tiffany et al., 2012; Marchand et al., 2019; Panlilio et al., 2020).

Another crucial need is increasing the number of **assessments and follow-up time-points**, as well as the **prospective length of time for post-treatment follow-ups**. Only a few studies assessed at a 12 months follow-up time-point or longer, thus prohibiting the examination of the long-term trajectory of abstinence, treatment outcomes, and recovery, which clearly awaits future research. Future studies employing multiple assessments over extended abstinence periods (>12 months) are warranted to more accurately capture the precise recovery time-course associated with treatment and protracted abstinence (or moderation) in individuals with SUD. Especially critical are EEG indicators at various phases related to SUD treatments (i.e., pre-SUD, pre-quit/pre-treatment baselines, detox/withdrawal, early/late abstinence, recovery, relapse, post-relapse).

Comprehensive SUD phenotyping can also play a crucial role in accurate identification of treatment outcomes and requires more **systematic assessment of non-EEG variables** including personality, family history, demographics, polysubstance use, psychiatric/medical comorbidities, and genetic profiles. Multivariate approaches, for example “deep phenotyping” (Lahnakoski et al., 2021; Phillips and Kendler, 2021) and subtyping (Hong et al., 2018), are being increasingly utilized in psychiatry (Beijers et al., 2019) and SUD (Kinreich et al., 2021) research. In the same vein, we also expect greater coordination (fusion) of EEG with SUD-relevant genomic (Huggett and Stallings, 2020), epigenetic (Farris and Mayfield, 2021), cardiovascular (Eddie et al., 2022), gastrointestinal (Meckel and Kiraly, 2019), metabolic (Caspani et al., 2022), and exposome (Pries et al., 2022) variables. Such deep phenotyping is also in line with multi-method/multi-level **transdiagnostic frameworks** such as NIMH Research Domain Criteria (Cuthbert, 2022). Addictions Neuroclinical Assessment factors (Kwako et al., 2017; Nieto et al., 2021), and Addiction Research Domain Criteria (Al-Khalil et al., 2021). Several past reviews (Houston and Schlienz, 2018; Jurado-Barba et al., 2020; Zhang et al., 2021b) summarized SUD-EEG links within these organizing frameworks, linking EEG/ERP metrics to *salience* (N1, P1, P3/SP, theta); *inhibitory control/conflict monitoring/executive* (ERN)/NG-N2/P3); and *negative emotionality/motivated attention*: (LPP, theta, face-elicited P1/N1/N170). Such system-level thinking, triangulating EEG/ERP metrics within broader nomothetic networks, will help future research better align with SUD-specific and transdiagnostic treatment models in psychiatry and medicine, leading to new knowledge of active ingredients in treatment-related behavior change (Houston and Schlienz, 2018; Rothman and Sheeran, 2021; Witkiewitz et al., 2022).

The **experimental EEG tasks** used across current studies are diverse and suggest a **need for common (standardized) task batteries**. Using both ERP-generating tasks (e.g., oddball, cue-reactivity) as well as resting EEG is recommended. Resting EEG data of up to 10 min are considered adequate for most analytic purposes in experimental, clinical and pharmaco-EEG (Jobert et al., 2012; Babiloni et al., 2020). In terms of tasks, we recommend using EEG task batteries, e.g., ERP CORE (Kappenman et al., 2021), and also at least two established tasks that have previously been used in several past SUD studies. The benefits of state-of-the-art task batteries are that each task is relatively brief (e.g., 10 min per ERP metric instead of 20–30 min) and leads to valid and reliable ERPs (Kappenman et al., 2021). The field needs to validate and use new tasks that are built to specifically access SUD-related cognition, e.g., mirroring clinical animal studies, focusing on features of SUD treatment such as craving, drug-seeking, personally meaningful stimuli, social environments and interpersonal cognition, and use of more naturalistic stimuli, i.e., gustatory or olfactory (Agarwal et al., 2021), music, movies. This need for SUD-specificity of tasks is in line with the current search for addiction-focused phenotype assessment batteries (Keyser-Marcus et al., 2021).

**Enhancing EEG assessment and analyses** can improve the validity, reliability, replicability, and overall transdiagnostic utility of SUD-EEG data. We recommend that future research studies adhere with up to date EEG guidelines for recording, analysis, and reporting (Keil et al., 2014, 2022; Clayson et al., 2019; Pernet et al., 2019; Babiloni et al., 2020; Styles et al., 2021) and leverage open-source data processing pipelines for replication and standardization (Pedroni et al., 2019; Desjardins et al., 2021; Delorme et al., 2022). Advances in the spatial resolution of distributed intracranial sources of EEG (Mahjoory et al., 2017; Samuelsson et al., 2021), and methods for indexing functional connectivity (Cao et al., 2022) and intrinsic networks (Sadaghiani et al., 2022), now allow for better linkages with transdiagnostic and multimodal neural circuit and brain-network findings. Current atlases of cognitive (Varoquaux et al., 2018; Bueichekú et al., 2020; Markello et al., 2022), neurochemical (Seidlitz et al., 2020) and neurogenetic (Seidlitz et al., 2020) distributions also allow for coordination of EEG data with multi-level brain function. Last, we noted a paucity of studies in our selected sample, relative to the broader field of EEG research, with analyses involving source estimation, functional and effective connectivity, event-related oscillations, single-trials, network or graph-theory, individualized frequency peak detection, and use of specific principal or independent components.

Finally, we emphasize that, relative to other brain imaging methods, high-quality EEG/ERP (and MEG) recordings *directly* index regional and global electrophysiological brain dynamics (e.g., neural activity, networks, and oscillations) with unparalleled millisecond temporal precision, providing both unique and complementary information about brain function. Further, although existing large-scale longitudinal or international multicenter studies are critical for enhancing knowledge, we recommend current and future mega-studies to also consider EEG as a brain imaging method, which, may allow for much higher sample sizes for the same costs as other brain imaging studies. Specifically, EEG equipment is much lower in cost than MRI and associated overheads, requires relatively brief training procedures, and is highly portable/mobile for deployment in medical centers, clinics, homes, and remote locations.

## Conclusion

The current scoping review included 44 empirical studies on EEG markers in abstinence and treatment studies. The main findings are that odd ball P3 and resting beta have strong evidence as useful potential biomarkers of SUD treatment outcomes, along with small but growing evidence for the utility of LPP, N2, delta, theta, and alpha, and for EEG effects from several forms of short-term treatment (stimulation, pharmacological, and behavioral). The use of EEG techniques in SUD abstinence and treatment research has increased substantially in the last two decades and these studies are instrumental in providing initial evidence that changes can occur longitudinally in ERP and spectral markers. Beyond providing hope for individuals with SUD and encouraging them to seek treatment, characterization of these neurobiological processes may help to identify novel EEG biomarkers that can be targeted for SUD interventions. Capturing the trajectory of neural changes over abstinence or long after treatment may help establish a neuroscience-informed framework for developing pharmacological, psychotherapeutic, and/or neuromodulatory interventions that can mimic and/or enhance the brain’s ability to repair itself, restoring cognitive function, and contributing to positive long-term treatment outcomes in individuals with SUD. Overall, SUD-EEG treatment studies require considerable investment of new research efforts, including more transparent and consistent reporting, longer abstinence and/or follow-up periods, larger sample sizes and deeper phenotyping, more focus on understudied SUDs, and enhancements in EEG tasks and analytic methods. Such advances will undoubtedly propel the use of EEG markers as effective biomarkers (e.g., prognostic, predictive, diagnostic, monitoring) for evidence-based treatments and precision psychiatry (García-Gutiérrez et al., 2020; Lahnakoski et al., 2021; Niculescu and Le-Niculescu, 2022).

## Author Contributions

TSB and MAP contributed equally in designing and writing the manuscript. AAK, TSB, and RBS contributed to literature search and review, risk of bias ratings, generation of tables, and content editing. All authors contributed to the article and approved the submitted version.

## Funding

This work was supported by grant funding to MAP from National Institute on Drug Abuse (K01DA043615).

## Abbreviations

AUD: Alcohol Use Disorder
CAN: Cannabis Use Disorder
CUD: Cocaine Use Disorder
dlPFC: Dorsolateral prefrontal cortex
EEG: Electroencephalography
ERN: Error-related negativity
ERP: Event-related potential
fMRI: Functional magnetic resonance imaging
Hz: Hertz
iRISA: impaired Response Inhibition and Salience Attribution
iTBS: intermittent theta burst stimulation
LPP: Late positive potential
mA: Milliampere
MDMA: 3,4-methylenedioxy-methamphetamine
MEG: Magnetoencephalography
MIX: Mixed, subsamples with several different SUDs
MMN: Mismatch Negativity
MUD: Methamphetamine Use Disorder
N2: Second negative peak in the averaged ERP waveform
NIC: Tobacco/Nicotine Use Disorder
OUD: Opioid Use Disorder
P3: Third positive component of an event-related potential
Pe: Error positivity
rTMS: repetitive Transcranial Magnetic Stimulation
SUD: Substance use disorders
T1: Time 1, usually first assessment
T2: Time 2, usually second assessment
tDCS: transcranial Direct Current Stimulation.
