# Supplementary material for: A scoping review of electroencephalographic (EEG) markers for tracking neurophysiological changes and predicting outcomes in substance use disorder treatment
Source: Front Hum Neurosci. 2022 Oct 17;16:995534. doi: 10.3389/fnhum.2022.995534 (PMC9619053; doi:10.3389/fnhum.2022.995534)
Supplement: Supplementary file 1 [file Data_Sheet_1.docx]

**Supplementary Table 1. Summary of other study characteristics**

| **First Author and Year** | **SUD and Study Type** | **Age** | **Sex** | **Education (years)** | **Ethnicity** | **Treatment / Abstinence**  **Time Details** | **Criteria / Interview** | **SUD Duration of Use (in years)** | **Usage Rate in SUD group** |
| --- | --- | --- | --- | --- | --- | --- | --- | --- | --- |
| **STUDIES OF ABSTINENCE & TREATMENT-AS-USUAL OUTCOMES** | | | | | | | | | |
| ***Event-related potentials (ERPs)*** | | | | | | | | | |
| Batschelet  2021 | AUD LONG | AUD: 43.47 (10.08) (24–60)  Controls: 44.70 (11.23) (27–58) | AUD: 38M/21F  Controls: 13M/7F | AUD:  14.03 (2.67) (9–20)  Controls: 15.25 (3.11) (12–23) | ** | Treatment:  8-12 weeks  Length ABST T1:  1-2 weeks  Length T2: 3 months | Criteria: DSM V diagnosis Interview: Yes*. | AUD:12.15 (8.92) (0–35) | ** |
| Campanella  2020 | AUD LONG | Relapse: 46 (11.2)  Abstinent: 49.3 (12.7) | Relapse: 20M/5F  Abstinent: 12M/3F | Relapse:  11 (3)  Abstinent: 12 (2) | ** | Treatment: 4 weeks  Length ABST T1:  4-7 days  Length ABST T2:  15-20 days  Relapse: 17.2 (1.7) days Abstinent: 16.4 (1.8) days  Length T3: 3 months | Criteria: DSM IV diagnosis Interview: No. | Relapse:16.2 (10.6)  Abstinent: 10.4 (10.1) | ** |
| Matheus-Roth  2016 | AUD LONG | AUD:  43.3 (9)  Controls:  44.3 (8) | AUD: 25M/6F  Controls:  25M/5F | AUD:  no formal education: 1  9-10 years: 14  10 years: 6  12-13 years: 9  Controls:  no formal education: 0  9-10 years: 9  10 years: 10  12-13 years: 12 | ** | Length ABST T1:  ≥ 10 days Length T2:  3 months | Criteria: DSM IV diagnosis Interview: Yes. | ** | ** |
| Petit  2014 | AUD LONG | AUD:  45 (12)  (20-68)  Controls:  45 (11)  (24-72) | AUD: 20M/7F  Controls: 20M/7F | AUD:  12 (3)  Controls:  16 (2) | ** | Length ABST T1:  3 weeks Length T2:  3 months | Criteria: DSM IV diagnosis Interview: No. | ** | Daily alcohol intake  AUD: 23 (14)  Controls: 0.5 (0.4) |
| Petit  2015 | AUD LONG | Relapse:  43 (11)  No-Relapse: 51 (12)  Controls:  44 (12) | Relapse: 14M/5F  No-Relapse: 14M/6F  Controls: 20M/9F | Relapse:  12 (3)  No-Relapse:  13 (3)  Controls:  16 (2) | ** | Treatment: 3 weeks  Length ABST T1:  Relapse: 18.7 (2.4) days  No-Relapse: 19.2 (1.7) days Length T2: 3 months | Criteria: DSM IV diagnosis Interview: No. | Relapse:  12 (10)  No-Relapse: 16 (9) | Daily alcohol intake  Relapse: 21 (12)  No-Relapse: 21 (16)  Controls: 0.5 (0.4) |
| Marhe  2013 | CUD LONG | CUD:  39.6 (8.4) Controls:  39.9 (9.4) | CUD: 89%M/11%F  Controls: 74%M/26%F | CUD: Primary School: 8, Junior School: 59, Senior School: 23, High School: 10; Controls: Primary School: 0, Junior School: 43, Senior School: 35, High School: 22 | ** | Treatment: 3 weeks  Length ABST T1:  3 days* Length T2:  3 months | Criteria: DSM IV diagnosis Interview: Yes. | 12.2 (6.8) | Cocaine used 17.6 (11.5) days of the last 30 days |
| Parvaz  2017 | CUD LONG | CUD: 41.6 (87.4) Controls:  43.20 (6.2) | CUD: 11M/8F  Controls: 13M/5F | CUD: 12.63 (2.9)  Controls: 13.08 (1.4) | ** | Length ABST T1: 143.88 (169.23) days  Length ABST T2: 183.12 (136.16) days | Criteria: DSM IV diagnosis Interview: Yes. | 14 (7.8) | days/week in the last 30 days  T1 0.53 (1.7) T2: 0.06 (0.2) |
| Lespine  2022 | NIC LONG | 43.3 (19-69) | 39M/81F | ** | ** | Length ABST T1:  prequit Length T2:  1 week  Length T3:  4 weeks* | Criteria: > than 5 cigarettes per day for last 12 months, Carbon Monoxide > than 5 parts per million Interview: Yes. | ** | at least 5 cigarettes/day for last 1 year |
| Luijten  2016 | NIC LONG | Relapse: 39.51 (16.07) (18–70)  No-Relapse: 46.56 (16.12) (16–68) | Relapse: 38%M/62%F  No-Relapse: 64%M/36%F | Relapse:  Low: 41 %, Medium: 32 %, High: 27%  No-Relapse: Low: 20%, Medium: 52%, High: 28% | ** | Length ABST T1:  1 hour Length T2:  4 weeks  Length T3:  8 weeks  Length T4:  12 weeks | Criteria: Carbon monoxide breath sample T1 Interview: No. | Relapse: 21.35 (16.04) (1–55)  No-Relapse: 25.27 (15.50) (0.50–53) | Relapse: 6.78 (0.68) (4–7) days/week  No-Relapse: 6.56 (1.16) days/week |
| Versace  2012 | NIC LONG | Cluster 1: 45.35 (10.7)  Cluster 2: 44.86 (10.3) | Cluster 1: 68M/31F  Cluster 2: 49M/32F | ** | Cluster 1: White: 70.7%  African-American: 23.2%  Other: 6.1%  Cluster 2: White: 65.4%  African-American: 32.1%  Other: 2.5% | Treatment:  10 weeks  Length ABST T1: 0  Length T2:  24 hours  Length T3:  4 weeks  Length T4:  6 weeks  Length T5:  10 weeks  Length T6:  12 weeks | Criteria: > than 5 cigarettes per day  Carbon Monoxide > than 6 parts per million Interview: No. | Cluster 1:  24.49 (12.1)  Cluster 2:  25.03 (11.1) | Cluster 1:  19.20 (8.4) cigarettes/day  Cluster 2:  18.99 (8.2) cigarettes/day |
| Haifeng  2015 | MUD LONG | MUD:  31.7 (7.5) Controls:  29.4 (6.6) | MUD: 14M/8F  Controls: 15M/14F | Junior High School & below:  MUD:17  Controls: 0  Senior High School & Above:  MUD: 7  Controls: 29 | Chinese: 100%* | Length ABST T1:  3-4 weeks  Length ABST T2:  3 months  Length ABST T3:  6 months | Criteria: DSM IV diagnosis Interview: Yes*. | 6.7 (2.8) | 50 g MA during the past 1 year |
| Lubman  2009 | OUD LONG | Relapse: 29.50 (6.6) (22-40)  No-Relapse: 32.89 (8) (22-46)  Controls: 30.0 (6.8) (19-46) | Relapse: 5M/7F  No-Relapse: 10M/9F  Controls: 9M/10F | ** | ** | Length ABST T1:  24 hours  Length T2:  6 (3.9-8) months | Criteria: DSM IV diagnosis Interview: Yes. | Relapse: 9.3 (5.3)  (1.3-18.9)  No-Relapse: 10.9 (6.4) (2.8-27.5) | ** |
| Anderson  2011 | MIX (CUD, MUD, OUD) LONG | Completers: 36.4 (11)  Discontinuers: 38.1 (11.6) | Completers: 77.3%M/22.7%F  Discontinuers: 76.9%M/23.1%F | Completers: 12.0 (2.9), Discontinuers: 15.0 (2.2) | Completers: White: 59.1%  Other: 40.9% Discontinuers:  White: 69.2%  Other: 30.8% | Treatment: **≥**3 months*  Length ABST T1:  **≥** 21 days Length ABST T2: Completers:  66.4 (40.5) days  Discontinuers:  48.4 (27.3) days | Criteria: DSM IV diagnosis Interview: Yes*. | ** | ** |
| Fink  2016 | MIX (CUD, MUD, OUD) LONG | Completers: 34.83 (8.76) Discontinuers: 34.2 (8.80) | Completers: 35M/63F  Discontinuers: 11M/14F | ** | White: 26%  Hispanic: 67%  African-American: 3%  Other: 4% | Treatment:  12 weeks  Length ABST T1:** Length ABST T2:  8-12 weeks* | Criteria: treatment seeking Interview: ** | Completers: 43.43 (23.36) months  Discontinuers: 49.27 (30.14) months | ** |
| Steele  2014 | MIX (CUD, MUD, OUD) LONG | 35.48 (8.47) | 34M/55F | ** | White: 28%, Hispanic: 65%  African-American: 2%  Other: 5% | Treatment:  12 weeks  Length ABST T1:** Length ABST T2:  8-12 weeks* | Criteria: treatment seeking Interview: ** | ** | ** |
| Wan  2010 | MIX (AUD, CUD, MUD, OUD, CAN)  LONG | Completers: 37.31 (11.41)  Discontinuers: 39.27 (11.33) | Completers: 80.77%M/19.23%F  Discontinuers: 80%M/20%F | Completers: 12.92 (2.24) Discontinuers: 13.53 (1.68) | Completers: White: 65.38%  Other: 34.62%; Discontinuers: White: 73.33%  Other: 26.67% | Treatment:  **≤** 6 months  Length ABST T1:  **≥** 21 days Length ABST T2: Completers:  66.04 (38.17) days  Discontinuers:  48.13 (25.64) days | Criteria: DSM IV diagnosis Interview: Yes. | ** | ** |
| ***Spectral Studies*** | | | | | | | | | |
| Januszko  2021 | AUD LONG | Abstinent: 48.5 (8.86)  Not-Abstinent: 44.63 (11.4) | 21M/13F  Abstinent: 13M/5F  Not-Abstinent: 9M/7F) | Abstinent:  12.00 (2.3)  Not-Abstinent:  12.19 (7.88) | White:100% | Length ABST T1:  Abstinent:  59.56 (33.9) days  Not-Abstinent:  53.12 (29.75) days  Length T2: median12 months (9-14) | Criteria: DSM IV diagnosis  Interview: Yes | Abstinent: 19.94 (10.73)  Not-Abstinent: 19.87 (11.86) | Abstinent: 415.03 (240.64)  Not-Abstinent: 550.57 (322.79) standard drinks in last 3 months  *[prior to T1]* |
| Saletu-Zyhlarz  2004 | AUD LONG | AUD: 41.5 (8.1)  Controls: 41.5 (8.4) | AUD: 15M/7F  Controls: 15M/7F | ** | ** | Length ABST T1:  14-42 days  Length T2: 6 months | Criteria:  DSM III R diagnosis Interview: ** | ** | ** |
| Levin  2007 | CUD LONG | 34.8 (4.1)  (23-39) | 16M/4F | ** | CUD: White:  15%  African-American: 80%  Asian/ Pacific Islander: 5% | Treatment: 3-13 weeks *  Length ABST T1:  16.8 (13.6) (0-48) days Length T2:  23.6 (14.8) (2–57) days  Length T3:  26 (16.6) (6-47) days | Criteria: DSM IV diagnosis  Interview: ** | 9 (5.4) | Cocaine used 21.4 (7.1) days of the last 30 days |
| Prichep  2002 | CUD LONG | 31.2  (17.7-47.3) | 41M/16F | ** | White:  8.8%  African-American: 70.2 %  Hispanic: 21.1% | Treatment: 18 months  Length ABST T1:  5-14 days  Length ABST T2:  25 weeks median | Criteria: DSM III-R diagnosis  Interview: Yes | Cluster 1: 8.9,  Cluster 2: 11.2  Cluster 3: 4.1 | ** |
| Venneman  2006 | CUD LONG | Completers: 33.2 (7)  Non-Completers: 31.4 (5) | Completers:  11M/3F  Non-Completers: 3M/2F | ** | ** | Length ABST T1:  10 days*  Length ABST T2:  ≤8 weeks | Criteria: DSM III R diagnosis Interview: Yes. | ** | ** |
| Allsop  2016 | CAN LONG | 21-36 | 6M/4F | ** | ** | Length ABST T1: prequit  Length ABST T2:  2 weeks | Criteria: DSM IV diagnosis Interview: Yes. | 7 to 18 | Prequit: 6.68 (3.91) (1.68-14 )  grams/week |
| Herning  2003 | CAN LONG | CAN: 32.6 (9.1)  Controls: 23.0 (5.1) | CAN: 80.7%M/19.3%F  Controls: 54.6%M/45.4%F | CAN:  12.0 (1.9)  Controls:  13.1 (1.8) | CAN: African-American: 83.9%  Other: 16.1%  Controls: African-American: 59.1%  Other: 40.9% | Length ABST T1:  72 hours  Length ABST T2:  28-30 days | Criteria: DSM III R diagnosis Interview: Yes. | CAN: 6.6 (3.8) | 24 (7.2) days in last 30 days |
| Herning  2008 | CAN LONG | CAN-short: 21.4 (3.4)  CAN-long: 24.3 (4.1)  Controls: 22.8 (5.3) | CAN-short: 67.7%M/32.3%F  CAN-long: 60.9%M/39.1%F  Controls: 47%M/53.0%F | CAN-short: 11.5 (1.4)  CAN-long: 12.0 (1.7)  Controls: 12.7 (1.3) | CAN-short: African-American: 86.2%  Other: 13.8%  CAN-long: African-American: 87%  Other: 13%  Controls: African-American: 75%  Other: 25% | Length ABST T1:  <72 hours  Length ABST T2:  1 month | Criteria: CAN use at least for 15 out of 30 days of a month Interview: Yes. | CAN-short: 4.4 (1.5)  CAN-long: 9.6 (1.8) | CAN-short: used 26.0 (5.1) days of the last 30 days  CAN-long: used 26.4 (4.9) days of the last 30 days |
| Bauer  2001b | MIX (AUD, CUD, OUD, benzodiazepines)  LONG | Relapse: 33.9 (6.4)  No-Relapse: 35.4 (6.6)  Controls: 35.6 (6.1) | Relapse: 76%M/24%F  No-Relapse: 62%M/38%F  Controls: 59%M/41%F | ** | No-Relapse: White: 44%  Other: 56%; Relapse: White: 45%  Other: 55%; Controls: White: 45%  Other: 55% | Length ABST T1*:  Relapse:  2.7 (1.2) months  No-Relapse:  2.9 (1.5) months  Length T2:  6 months | Criteria: DSM III R diagnosis Interview: Yes. | ** | ** |
| **TREATMENT STUDIES** | | | | | | | | | |
| ***Brain Stimulation*** | | | | | | | | | |
| da Silva  2013 | AUD Pre-Post STIM | Sham:  49 (29-59)  Active:  49 (40-52) | 98M/0F | 0–4:  Sham: 4 (57%)  Active: 3 (50%) 5–8:  Sham: 2 (27%)  Active: 0 (0%) 9–12:  Sham: 0 (0%)  Active: 3 (50%) >13:  Sham: 1 (14%)  Active: 0 (0%) | ** | Treatment:  5 weeks  Length ABST T1:  Sham: 10 (1-34) days, Active: 6 (1-29) days  Length T2:  5 weeks  Length T3:  4 weeks* | Criteria: DSM-IV diagnosis  Lesch type IV Interview: Yes. | ** | Sham:  148 (25-291) grams/day  Active: 330.5 (93-370) grams/day |
| Del Felice  2016 | AUD Pre-Post STIM | Sham:  43.2 (10.2)  Active:  45.6 (8.4) | Sham:  6M/3F  Active: 7M/1F | Sham:  12.0 (2.7)  Active:  8.9 (2.7) | ** | Treatment:  2 weeks  Length ABST T1:  ≥ 9 days  Length ABST T2:  4 weeks | Criteria: DSM IV diagnosis Interview: Yes. | ** | ** |
| Nakamura-Palacios  2012 | AUD Pre-Post STIM | Lesch I:  48.8 (10.7)  Lesch II:  50.1 (8.4)  Lesch III:  49.7 (7.1)  Lesch IV:  46.8 (9.4) | Lesch I: 16M/0F  Lesch II: 7M/0F  Lesch III: 12M/2F  Lesch IV: 10M/2F | Elementary School:  Lesch I:85.7%  Lesch II:100%  Lesch III: 83%  Lesch IV: 90%  High School:  Lesch I: 7%  Lesch II: 0%  Lesch III: 17%  Lesch IV:10%  Higher Education: Lesch I: 7%  Lesch II: 0%  Lesch III: 0%  Lesch IV: 0% | ** | Length ABST T1:  ≥7 days  Length T2:  3 weeks* | Criteria: ICD-10 diagnosis Interview: Yes. | ** | Lesch I:  21.5 (20.6) drinks/day  Lesch II:  7.2 (2.6) drinks/day  Lesch III:  12.1 (8.0) drinks/day  Lesch IV:  22.0 (19.3) drinks/day |
| Naim-Feil  2022 | AUD  Pre-Post  STIM | AUD:  40 (14)  Controls:  32 (6) | AUD:  7M/4F  Controls:  8M/8F | ** | ** | Length ABST T1:  39 Median days  (8–668) | Criteria: DSM IV diagnosis  Interview: Yes | ~2 years post-treatment and self-reported abstinent in last month |  |
| Conti  2014a | CUD (crack) Pre-Post STIM | Sham:  27.5 (5.3) Active:  32 (8) | Sham:  5M/1F  Active:  6M/1F | 5–8 years: Sham: 3  Active: 2  9–12 years: Sham: 3  Active: 4  >13 years: Sham: 0  Active: 1 | ** | Treatment:  12 days  Length ABST at T1:  Sham: 9.2 (10.2) days  Active: 22.1 (30.5) days | Criteria: DSM IV diagnosis Interview: Yes. | ** | Sham:  115 (133) rocks/week  Active: 125 (104.4) rocks/week |
| Conti  2014b | CUD (crack) Pre-Post STIM | 30 (7) | ** | ** | ** | Treatment:  20 minutes  Length ABST T1:  16 (23) days | Criteria: DSM IV diagnosis Interview: ** | ** | ** |
| Pripfl  2014 | NIC Pre-Post STIM | 29.2 (5.5)  (21-38) | 5M/6F | ** | ** | Length ABST T1:  ≥6 hours  Length ABST T2:  ≥6 hours | Criteria: ICD-10 diagnosis Interview: No. | Smoked for at least 1 year* | 10 cigarettes/day for at least 1 year |
| Chen  2021 | MUD Pre-Post STIM | Sham:  30.73 (6.68)  Active:  29.66 (4.70) | Sham:  11M/10F  Active:  20M/10F | Sham:  9.05 (2.36)  Active:  8.69 (2.39) | ** | Treatment:  4 weeks  Length ABST T1:  Sham: 3.05 (1.40) months Active: 2.63 (1.42) months  Length ABST T2:  +4 weeks* | Criteria: DSM IV diagnosis Interview: Yes. | Sham:  5.00 (3.89)  Active: 5.00 (3.21) | ** |
| Khajehpour  2022 | MUD Pre-Post STIM | Sham:  32.1 (1.6)  Active:  33.3 (1.7) | 42M/0F | Sham:  112.3 (3.3  Active:  14.5 (3) | 100% Persian* | Length ABST T1:  1 week to 6 months | Criteria: DSM V diagnosis Interview: No | Sham:  7.1 (0.98) Active: 8.03 (1.1) | ** |
| Zhang  2021 | MUD Pre-Post STIM | Sham:  31.6 (4.5)  Active:  31.5 (5.1) | 40M/0F | Sham:  8.9 (2)  Active:  8.6 (1.9) | 100% Chinese* | Treatment:  10 days  Length ABST T1:  1 day*  Length ABST T2:  Sham: 38.20 (32.32)  Active: 47.89 (55.09) | Criteria: DSM V diagnosis.  Interview: No | Sham:  8.96 (6.44)  Active:  6.52 (3.67) | Sham: 8.97 (8.83) grams/month Active: 13.44 (12.09) grams/month |
| Mostafavi  2022 | OUD Pre-Post STIM | Group A: 34.40 (7.66)  Group B: 32.27 (9.99)  Sham: 32.70 (9.34) | 30M/0F | Masters:  Group A: 4  Group B: 7  Sham: 4 High School:  Group A: 2  Group B: 0  Sham: 2 Diploma:  Group A: 3  Group B: 3  Sham: 4 Associates Degree:  Group A: 1  Group B: 0  Sham: 0 | ** | Treatment:  10 days  Length T1: ≥ 10 days Methadone treatment | Criteria: DSM IV diagnosis Interview: Yes. | Group A: 11.90 (4.93)  Group B: 12.36 (5.76)  Sham: 12.50 (6.58) | ** |
| Nakamura-Palacios  2016 | MIX (AUD,CUD) Pre-Post STIM | AUD:  Sham: 46.8 (7)  Active: 41.4 (4)  CUD:  Sham:  29.7 (6.7)  Active: 30.8 (8.1) | AUD:  Sham: 13M/1F  Active: 8M/0F  CUD:  Sham: 3M/0F  Active: 6M0F | ** | ** | Treatment:  5-10 days*  Length ABST T1: ** | Criteria: DSM IV and ICD-10 diagnosis Interview: Yes | ** | AUD:  Sham: 18.7 (15.8) drinks/day  Active:  17 (15.8) drinks/day  CUD:  Sham: 17.7 (26.3) rocks/day  Active: 20.8 (15.9) rocks/day |
| ***Pharmacological and Behavioral Treatments*** | | | | | | | | | |
| Brown  2020 | AUD Pre-Post BEH+ STIM | MBRP+  Active-tDCS: 50.89 (14.85) MBRP+  Sham-tDCS: 53.59 (12.04) | MBRP+  Active-tDCS: 18M/18F  MBRP+  Sham-tDCS: 18M/14F | ** | White: 50%, Hispanic: 39.7%  Other: 11.3% | Treatment:  2-3 months  Length ABST T1:  24 hours* Length ABST T2:  24 hours* | Criteria: DSM V diagnosis  Interview: Yes. | ** | MBRP+  Active-tDCS:3.65 (2.49) drinks/day  MBRP+  Sham-tDCS: 4.22 (3.80) drinks/day |
| Martinez-Maldonado  2020 | AUD Pre-Post BEH | A-CBM: 53 (9.80)  N-CBM: 54.25 (7.04)  No-Intervention: 53.91 (2.03) | A-CBM:  9M/1F  N-CBM: 11M/1F  No-Intervention: 10M/1F | Primary:  A-CBM: 20%  N-CBM: 50%  No-Intervention: 27.3%  Secondary:  A-CBM: 10%  N-CBM: 16.7%  No-Intervention:  136.4%  University:  A-CBM: 40%  N-CBM: 8.3%  No-Intervention: 18.2% | ** | Treatment:  1 week  Length ABST T1:  A-CBM: 7.30 (3.86) days  N-CBM: 4.50 (2.39) days  No-Intervention:  6.45 (2.54) days  Length ABST T2:  +1 week | Criteria: DSM V diagnosis  Interview: Yes*. | A-CBM: 17.10 (13.76)  N-CBM: 14.08 (10.54)  No-Intervention: 13.64 (12.14) | ** |
| Cinciripini  2017 | NIC Pre-Post Drug | Varenicline: 43.3 (11.2)  Bupropion: 46 (9.01)  Placebo: 46 (11.1) | Varenicline: 36M/22F  Bupropion: 42M/17F  Placebo: 39M/24F | ** | White/ African-American/ Hispanic/ Other:  Varenicline:  52/27.6/17.2/6.9%  Bupropion: 67.5/ 27.1/6.8/5.1%  Placebo: 64.7/27/ 7.9/1.6% | Treatment:  12 weeks  Length ABST T1: prequit  Length ABST T2:  12 weeks  Length T3:  3 months  Length T4:  6 months | Criteria: DSM IV diagnosis  Interview: Yes. | ** | Varenicline: 18.9 (8.9) cigarettes/day  Bupropion: 19.3 (7.2) cigarettes/day  Placebo: 18.6 (8.7) cigarettes/day |
| Li  2017 | NIC Pre-Post BEH | 26 (5.9) (20-48) | 42M/0F | ** | ** | Treatment:  1 hour | Criteria: FTND Interview: No. | 7.2 (4.8) | 15 (4.7) cigarettes/day  for ≥ 2 years |
| Macatee  2022 | CAN Pre-Post BEH | DTI: 20.37 (2.95)  HVC: 20.17 (1.63) | DTI: 30%M/66.67%F/ 3.33% other  HVC: 31.03%M/65.52%F/ 3.45% other | ** | ** | Treatment:  2 sessions over 1 week  Length ASBT T1/T2:  ≥24 hours  Length T1: baseline  Length T2: 2 week Length T3: 1 month  Length T4: 4 months | Criteria:  Self-Reported cannabis use ≥ 2 times per week for ≥ 1 year Interview: Yes | DTI:  3.77 (2.67)  HVC: 3.12 (1.47) | DTI:  21.73 (6.35) days of the last month  HVC:  20 (5.87) days of the last month |
| Motlagh  2018 | OUD Pre-Post Drug | OUD:  36.8 (6.6)  Controls:  38.9 (11.4) | 54M/0F | OUD:  3.3 (0.6)  Controls:  11.9 (4.3) | ** | Length ABST T1:  1 week  Length ABST T2:  10 weeks | Criteria: DSM IV diagnosis  Interview: Yes: | 19.1 (5.9) | ** |
| Robinson  2022 | MIX (NIC, AUD) Pre-Post Drug | Placebo: 46.9 (9.7)  Topiramate-low: 46.4 (10.7)  Topiramate-high: 47.1 (11.9) | Placebo: 26M/10F  Topiramate-low: 19M/10F  Topiramate-high: 25M/11F | ** | Placebo: White: 50%, African-American: 36.1%  Other: 13.9%; Topiramate-low: White:41.4%  African-American: 48.3%  Other: 6.9%; Topiramate-high:  White: 61.1%, African-American: 33.3%  Other:  2.8% | Treatment:  18 weeks, abstinence started at 5th week  Length T1: baseline Length T2: 5 weeks  Length T3: 1 month  Length T4: 3 months | Criteria: DSM V diagnosis  Interview: Yes | ** | Topiramate-low:  9.2 (6.7) drinks/day  18.1 (9.5) cigarettes/day  Topiramate-high: 8.5 (5.7) drinks/day  19.8 (8.0) cigarettes/day  Placebo: 7.8 (5.1) drinks/day  18.6 (8.6) cigarettes/day |

**Supplementary Table 2.** Tool 1 quality/bias questions: Quality Assessment Tool for Before-After (Pre-Post) Studies with No Control Group

| 1. Was the study question or objective clearly stated? |
| --- |
| 2. Were eligibility/selection criteria for the study population prespecified and clearly described? |
| 3. Were the participants in the study representative of those who would be eligible for the test/service/intervention in the general or clinical population of interest? |
| 4. Were all eligible participants that met the prespecified entry criteria enrolled? |
| 5. Was the sample size sufficiently large to provide confidence in the findings? |
| 6. Was the test/service/intervention clearly described and delivered consistently across the study population? |
| 7. Were the outcome measures prespecified, clearly defined, valid, reliable, and assessed consistently across all study participants? |
| 8. Were the people assessing the outcomes blinded to the participants' exposures/interventions? |
| 9. Was the loss to follow-up after baseline 20% or less? Were those lost to follow-up accounted for in the analysis? |
| 10. Did the statistical methods examine changes in outcome measures from before to after the intervention? Were statistical tests done that provided p values for the pre-to-post changes? |
| 11. Were outcome measures of interest taken multiple times before the intervention and multiple times after the intervention (i.e., did they use an interrupted time-series design)? |

**Supplementary Table 3.** Tool 2 quality/bias questions: Quality Assessment of Controlled Intervention Studies

| 1. Was the study described as randomized, a randomized trial, a randomized clinical trial, or an RCT? |
| --- |
| 2. Was the method of randomization adequate (i.e., use of randomly generated assignment)? |
| 3. Was the treatment allocation concealed (so that assignments could not be predicted)? |
| 4. Were study participants and providers blinded to treatment group assignment? |
| 5. Were the people assessing the outcomes blinded to the participants' group assignments? |
| 6. Were the groups similar at baseline on important characteristics that could affect outcomes (e.g., demographics, risk factors, co-morbid conditions)? |
| 7. Was the overall drop-out rate from the study at endpoint 20% or lower of the number allocated to treatment? |
| 8. Was the differential drop-out rate (between treatment groups) at endpoint 15 percentage points or lower? |
| 9. Was there high adherence to the intervention protocols for each treatment group? |
| 10. Were other interventions avoided or similar in the groups (e.g., similar background treatments)? |
| 11. Were outcomes assessed using valid and reliable measures, implemented consistently across all study participants? |
| 12. Did the authors report that the sample size was sufficiently large to be able to detect a difference in the main outcome between groups with at least 80% power? |
| 13. Were outcomes reported or subgroups analyzed prespecified (i.e., identified before analyses were conducted)? |
| 14. Were all randomized participants analyzed in the group to which they were originally assigned, i.e., did they use an intention-to-treat analysis? |

**Supplementary Table 4.** Responses: Quality Assessment Tool for Before-After (Pre-Post) Studies with No Control Group

|  | Q1 | Q2 | Q3 | Q4 | Q5 | Q6 | Q7 | Q8 | Q9 | Q10 | Q11 | Overall |  |
| --- | --- | --- | --- | --- | --- | --- | --- | --- | --- | --- | --- | --- | --- |
| Allsop 2016 | Y | Y | Y | NR | N | Y | Y | N | Y | Y | N | F | **78** |
| Anderson 2011 | Y | Y | Y | NR | N | Y | Y | N | Y | N | N | G | **67** |
| Batschelet 2021 | Y | Y | Y | NR | Y | Y | Y | N | Y | N | N | G | **78** |
| Bauer 2001b | Y | Y | Y | NR | Y | Y | Y | N | Y | N | N | G | **78** |
| Campanella 2020 | Y | Y | Y | NR | N | Y | Y | N | Y | Y | N | G | **78** |
| Fink 2016 | Y | Y | Y | NR | Y | Y | Y | N | Y | N | N | G | **78** |
| Haifeng 2015 | Y | Y | Y | NR | N | Y | Y | N | Y | Y | N | G | **78** |
| Herning 2008 | Y | Y | Y | NR | Y | Y | Y | N | N | N | N | G | **67** |
| Herning 2003 | Y | Y | Y | NR | Y | N/CD | Y | N | Y | N | N | F | **67** |
| Januszko 2021 | Y | Y | Y | NR | N | Y | Y | N | N | N | N | G | **56** |
| Lespin 2022 | Y | Y | Y | NR | Y | Y | Y | Y | Y | Y | N | G | **100** |
| Levin 2007 | Y | Y | Y | NR | N | N/CD | Y | N | N | N | N | F | **44** |
| Li 2009 | Y | N | Y | NR | Y | Y | Y | N | N/NA | Y | N | F | **67** |
| Lubman 2016 | Y | Y | Y | NR | N | Y | Y | N | Y | N | N | G | **67** |
| Luijten 2013 | Y | Y | Y | NR | N | Y | Y | N | Y | Y | N | G | **78** |
| Marhe 2016 | Y | Y | Y | NR | N | Y | Y | N | Y | N | N | G | **67** |
| Matheus-Roth 2018 | Y | Y | Y | NR | N | Y | Y | N | Y | N | N | G | **67** |
| Motlagh 2017 | Y | Y | Y | NR | N | Y | Y | N | N | Y | N | G | **67** |
| Naim-Fell 2014 | Y | Y | Y | NR | N | Y | Y | N | N/NA | Y | N | G | **67** |
| Parvaz 2015 | Y | Y | Y | NR | N | Y | Y | N | Y | Y | N | G | **78** |
| Petit 2002 | Y | Y | Y | NR | N | Y | Y | N | Y | N | N | G | **67** |
| Petit 2004 | Y | Y | Y | NR | N | Y | Y | N | Y | N | N | G | **67** |
| Prichep 2013 | Y | Y | Y | NR | N | Y | Y | N | N | N | N | G | **56** |
| Pripfl 2012 | Y | Y | Y | NR | N | Y | Y | N | Y | Y | N | G | **78** |
| Saletu-Zyhlarz 2010 | Y | Y | Y | NR | N | N/CD | Y | N | Y | N | N | F | **56** |
| Steele 2006 | Y | Y | Y | NR | Y | Y | Y | N | Y | N | N | G | **78** |
| Venneman 2022 | Y | Y | Y | NR | N | N/CD | Y | N | N/NA | N | N | F | **44** |
| Versace 2017 | Y | Y | Y | NR | Y | Y | Y | N | N | N | N | G | **67** |
| Wan 2014 | Y | Y | Y | NR | N | Y | Y | N | Y | N | N | G | **67** |
|  | **100** | **97** | **100** | **0** | **31** | **86** | **100** | **3** | **69** | **34** | **0** | **79** | **69** |

**Supplementary Table 5.** Responses: Quality Assessment of Controlled Intervention Studies

|  | Q1 | Q2 | Q3 | Q4 | Q5 | Q6 | Q7 | Q8 | Q9 | Q10 | Q11 | Q12 | Q13 | Q14 | Overall |  |
| --- | --- | --- | --- | --- | --- | --- | --- | --- | --- | --- | --- | --- | --- | --- | --- | --- |
| Brown  2020 | Y | Y | Y | Y | N | Y | Y | Y | Y | Y | Y | N | Y | NR | G | **79** |
| Chen  2021 | Y | Y | Y | N | Y | Y | Y | Y | Y | Y | Y | N | Y | NR | G | **79** |
| Cinciripini  2017 | Y | Y | Y | Y/N | NR | Y | Y | Y | Y | Y | Y | N | Y | NR | G | **71** |
| Conti  2014a | Y | Y | N | N | NR | Y | N | N | N | Y | Y | N | Y | NR | F | **43** |
| Conti  2014b | Y | NA | NR | NR | NR | NA | Y | NA | N | Y | Y | N | Y | NR | F | **36** |
| da Silva 2013 | Y | Y | Y | N | N | Y | Y | Y | Y | Y | Y | N | Y | NR | G | **71** |
| Del Felice  2016 | Y | Y | Y | N | N | Y | Y | Y | Y | Y | Y | N | Y | NR | G | **71** |
| Khajehpour  2022 | Y | Y | Y | NR | Y | Y | Y | Y | Y | Y | Y | Y | Y | NR | G | **79** |
| Macatee  2022 | Y | Y | N | N | N | Y | N | Y | Y | Y | Y | Y | Y | NR | G | **64** |
| Martinez-Maldonado  2020 | Y | Y | NR | NR | NR | Y | Y | Y | Y | Y | Y | N | Y | NR | G | **64** |
| Mostafavi  2006 | Y | Y | Y | Y | Y | Y | Y | Y | Y | Y | Y | N | Y | NR | G | **86** |
| Nakamura-Palacios  2016 | Y | Y | Y | N | N | Y | Y | Y | Y | Y | Y | N | Y | NR | G | **71** |
| Nakamura-Palacios  2012 | N | Y | NR | N | NR | N | Y | Y | Y | Y | Y | N | Y | NR | G | **50** |
| Robinson  2022 | Y | Y | Y | Y | Y | N | N | Y | Y | Y | Y | N | Y | NR | G | **71** |
| Zhang  2021 | Y | Y | Y | N | N | Y | N | Y | Y | Y | Y | N | Y | NR | G | **64** |
|  | **93** | **93** | **67** | **20** | **20** | **80** | **73** | **87** | **87** | **100** | **100** | **13** | **100** | **0** | **87** | **67** |
